# Neuroimaging PheWAS and molecular phenotyping implicate *PSMC3* in Alzheimer's disease

**DOI:** 10.1002/alz.71217

**Published:** 2026-02-24

**Authors:** Xavier Bledsoe, Ting‐Chen Wang, Yiyang Wu, Derek Archer, Hung Hsin Chen, Adam C. Naj, William S. Bush, Timothy J. Hohman, Logan Dumitrescu, Jennifer E. Below, Eric R. Gamazon

**Affiliations:** ^1^ Vanderbilt Genetics Institute Vanderbilt University Medical Center Nashville Tennessee USA; ^2^ Vanderbilt Memory and Alzheimer's Center Vanderbilt University Medical Center Nashville Tennessee USA; ^3^ Institute of Biomedical Sciences Academia Sinica Taipei Taiwan; ^4^ Department of Biostatistics Epidemiology, and Informatics Perelman School of Medicine, University of Pennsylvania Philadelphia Pennsylvania USA; ^5^ Penn Neurodegeneration Genomics Center Department of Pathology and Laboratory Medicine Perelman School of Medicine University of Pennsylvania Philadelphia Pennsylvania USA; ^6^ Department of Population and Quantitative Health Sciences Cleveland Institute for Computational Biology Case Western Reserve University School of Medicine Cleveland Ohio USA

**Keywords:** Alzheimer's disease, dementia family history, genetic covariance, NeuroimaGene, neuroimaging‐derived phenotypes, transcriptome‐wide association studies

## Abstract

**INTRODUCTION:**

Neuroimaging genetics has advanced our understanding of Alzheimer's disease (AD); however, frameworks using functional genomics are needed to elucidate mechanisms connecting loci to neurological outcomes. To address this need, we explored relationships between AD‐associated variants and disease via their impact on gene expression and neuroanatomical phenotypes.

**METHODS:**

We mapped established AD genes to neuroimaging traits using the NeuroimaGene Atlas and predicted transcript‐driven neurological features of AD by comparing gene‐derived neuroimaging features with clinical neuroimaging data. Genetic covariance analyses were performed to characterize shared genetic architecture between AD endophenotypes and neuroimaging features, and to identify neuroimaging features associated with a family history of dementia.

**RESULTS:**

Our analyses implicate *PSMC3* as a contributor to AD pathophysiology and identify AD endophenotypes, including dementia family history, linked to frontal cortex thickness and volume, as well as changes in cerebrospinal fluid volume.

**DISCUSSION:**

Our findings prioritize AD genes whose regulation is associated with vulnerable brain regions, offering a potential mechanistic framework for downstream functional validation.

## Highlights

- Alzheimer's disease (AD) genes relate to brain regions key to memory and cognition via genetic regulation of expression.
- *PSMC3*, involved in protein degradation, is linked to AD and brain morphology.
- Increased *PSMC3* levels associate with better cognition and lower amyloid beta levels.
- Genetic covariance shows shared genetic architecture between hippocampus traits and AD risk.
- AD‐associated genetic variants affect the frontal cortex, cerebrospinal fluid volume, and cingulate isthmus gyrus.

## 1 BACKGROUND

Alzheimer's disease (AD) is a complex polygenic neurodegenerative disorder with an estimated heritability (*h^2^*) of 60% to 80%.^1^, ^2^ This high heritability provides an opportunity to leverage genetic studies in characterizing the molecular basis of AD risk. Genome‐wide association studies (GWASs) of AD and AD‐related phenotypes, including AD clinical diagnosis^3^, ^4^ and parental dementia status (AD by proxy),^5^ and rare variant analyses^6^ have all identified a host of genetic variants associated with AD risk. Investigating the functional implications of these variants is important both to advance our comprehension of the mechanisms underlying AD etiology and to facilitate the development of innovative therapeutic interventions.

Neuroimaging genomics has produced a range of approaches for integrating single‐nucleotide polymorphism (SNP) and gene‐level associations with neuroimaging data to investigate how genetic variation influences brain structure and function in the context of disease development. Prominent SNP‐driven approaches include BrainXcan,^7^ brain‐wide GWASs,^8^ and univariate and multivariate image‐wide association studies (IWAS).^9^, ^10^ However, the use of SNPs as predictor variables in neuroimaging genetics research poses limitations. One main challenge is that GWAS‐identified SNPs are often located in non‐coding regions, limiting the ability to interpret their direct biological implications.^11^ This limitation underscores the utility of approaches such as transcriptome‐wide association study (TWAS). TWAS is a multivariate approach that uses expression quantitative trait loci (eQTL) data to identify associations between genetically regulated gene expression (GReX) and a trait of interest.^12^ TWAS extends GWAS by combining GWAS summary statistics with eQTL reference data. This strategy enables TWAS to identify gene–trait links in loci that might not reach genome‐wide statistical significance in GWAS and thus might otherwise be overlooked. Furthermore, TWAS reduces the multiple testing burden by testing at the gene level rather than at the variant level, enhancing statistical power and yielding more interpretable, gene‐based results than GWAS in a disease context.^13^ Applied to AD, TWAS enables the mapping of non‐coding risk SNPs to genes through GReX. These genes can then be aggregated as a transcriptomic signature and analyzed for mechanistic insight.^14^, ^15^ While TWAS of AD and AD‐associated phenotypes have been performed,^16^, ^17^, ^18^ the relationship of AD GReX to neuroimaging‐derived phenotypes remains an underexplored subject.

**FIGURE 1 alz71217-fig-0001:**
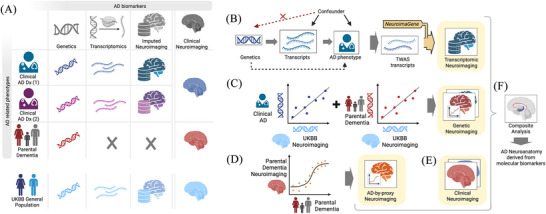
Schematic overview of the analytical framework. A, Grid summarizing primary data resources integrated in the study. B, Directed acyclic graph illustrating TWAS analyses and downstream imputation of neuroimaging features via NeuroimaGene. C, Visualization of genetic covariance analyses comparing the genetic architecture of clinical AD and parental AD with neuroimaging‐derived features. D, Logistic regression models evaluating associations between neuroimaging features and parental AD status. E, Integration of clinical neuroimaging data linking brain features to AD status. F, Composite synthesis comparing the neuroimaging features obtained across transcriptomic, genetic covariance, parental history, and clinical approaches. AD, Alzheimer's disease; Dx, diagnosis; TWAS, transcriptome‐wide association study; UKBB, UK Biobank.

In this study, we investigated the relationship between molecular endophenotypes of AD and neuroimaging features (Figure 1A and Figure S1 in supporting information). The recently published NeuroimaGene resource used TWAS to identify associations between tissue‐specific GReX and neuroimaging‐derived phenotypes (NIDPs) generated from > 33,000 study participants in the UK Biobank (UKB).^19^, ^20^, ^21^, ^22^ We leveraged NeuroimaGene to characterize the effect of AD TWAS genes on the structure and function of the brain (Figure 1B) using two complementary analytical frameworks. Initially, a hypothesis‐driven targeted analysis tested associations between AD TWAS genes and neuroanatomical regions observed to differ by AD status based on clinical studies.^23^, ^24^ Second, a hypothesis‐free, image‐agnostic approach was designed to discover novel neuroanatomical correlates of AD TWAS genes.^3^ Complementing these analyses, we also investigated genome‐wide and local genetic covariance between brain morphology and established AD GWAS profiles for AD diagnosis and parental dementia (Figure 1C). Furthermore, to validate and substantiate the clinical relevance of these genetic findings at the phenotypic level, we evaluated direct phenotypic correlation between NIDPs and family history of dementia (Figure 1D). Taken together, neuroimaging findings derived from genetic, transcriptomic, and covariance analyses in relation to AD risk genetic architecture provide a critical framework for elucidating the neurological mechanisms leading to disease risk (Figure 1E,F).

## 2 METHODS

Details about each data source used in this study are provided in Table S1 in supporting information.

### 2.1 Genetic regulation of AD gene expression for previously identified genes

We initially identified 32 genes of interest reported by Chen et al.^3^ whose GReX was associated with AD disease status in the Alzheimer's Disease Genetic Consortium (ADGC) and International Genomics of Alzheimer's Project (IGAP) consortia. The MetaXcan cross‐tissue TWAS framework used gene expression models trained exclusively on brain tissues to determine these associations.^23^ Following causal inference via Mendelian randomization (MR) in their study, 23 of the gene‐level associations retained statistical significance. We therefore included the GReX of these 23 genes as input variables in the image‐agnostic and image‐directed analyses described below.

### 2.2 Curation of AD‐associated neuroimaging features for image‐directed analysis

To facilitate hypothesis‐driven NeuroimaGene analysis, we leveraged clinically relevant imaging measures identified by Westman et al.^24^ In that study, magnetic resonance imaging (MRI) scans of 699 US and Canadian participants categorized as AD cases, cognitively intact controls, or individuals with mild cognitive impairment (MCI) from the Alzheimer's Disease Neuroimaging Initiative (ADNI) were analyzed, and brain measures that differed by cognitive status were identified. Importantly, their study applied the same image‐processing pipeline and parcellation and segmentation procedures (detailed below) as those used in UKB. The methodological alignment ensured comparability and consistency in our examination of associations with these imaging measures.

**RESEARCH IN CONTEXT**

1. **Systematic review**: We systematically reviewed Alzheimer's disease (AD) imaging genetics research using sources such as PubMed and Google Scholar. Although prior research has linked AD risk variants to imaging traits, comprehensive analyses using large‐scale, mostly non‐AD imaging cohorts that integrate genetic, transcriptomic, and epidemiological risk evaluations remain limited.
2. **Interpretation**: We found enrichment of AD risk gene associations with neuroimaging‐derived phenotypes in the frontal cortex, cerebrospinal fluid volume, and the isthmus of the cingulate gyrus using multiple analytic strategies. Notably, *PSMC3* expression demonstrated the most pronounced association with ventral diencephalon volume, a finding that was consistent across both disease‐agnostic and clinically informed analyses.
3. **Future directions**: Our study highlights the utility of linking AD risk genes to brain anatomical phenotypes for prioritizing gene targets. Future research should investigate longitudinal gene expression changes and their impact on regional brain structures and pathology in diverse populations to better clarify causal roles in AD progression and refine target prioritization.

Westman et al. applied the FreeSurfer pipeline uniformly to scans from all patients to generate neuroimaging‐derived phenotypes corresponding to regional volume, cortical volume, surface area, and thickness. The atlases used for[Fig alz71217-fig-0001] the parcellation of cortical and subcortical regions include the Destrieux atlas,^25^ the Desikan atlas,^26^ and the initial subcortical atlas reported in Fischl et al.^27^ Using these data, they generated a prediction model trained on the neuroimaging measures for discriminating between patients with and without AD. The model includes the covariance of each neuroimaging measure with disease status as well as the confidence interval. We prioritized the 59 neuroimaging measures that demonstrate covariance with disease status and do not include zero within the confidence interval. Of note, the NIDPs reported by Westman et al. reflect averages across the left and right hemispheres, whereas the UKB reported hemisphere‐specific measurements. For each significant covarying NIDP in Westman et al., we selected the corresponding regions in both the left and right hemispheres aligned by the FreeSurfer segmentation atlas. Control for multiple testing is described below.

For this analysis, we used the NeuroimaGene resource described in Bledsoe et al.^20^ Of the 59 NIDPs reported by Westman et al., 57 are available in the NeuroimaGene resource, with cortical measures represented separately for the left and right hemispheres.^20^ The two missing phenotypes resulted from a lack of significant SNP‐based heritability and failure to pass quality control (QC) during the neuroimaging phase of the UKB data generation. Including cortical regions duplicated in the right and left hemispheres, we selected a total of 110 NIDPs as AD‐informative phenotypes for the image‐directed analyses.

### 2.3 Multiple testing correction for the image‐directed analysis

Previous work demonstrates that gene expression is highly correlated and non‐independent across tissue contexts.^28^ Therefore, we relied on the study‐wide Benjamini–Hochberg false discovery rate (FDR) threshold of 0.05.^29^

### 2.4 Gene expression in Religious Orders Study and Memory and Aging Project bulk RNA sequencing and AD endophenotypes

We used processed bulk RNA sequencing (RNAseq) data from three brain tissues in the Religious Orders Study and Memory and Aging Project (ROSMAP) cohort, including dorsolateral prefrontal cortex (DLPFC, *N* = 208), posterior cingulate cortex (PCC, *N* = 490), and the head of the caudate nucleus (CN, *N* = 673)^30^. These data were used to examine whether expression of genes from image‐directed analyses with causal evidence was associated with AD endophenotypes, including AD pathology, amyloid beta (Aβ) load (immunohistochemistry [IHC] staining), neurofibrillary tangles (IHC and silver staining), and cognitive function via performing multiple cross‐sectional and longitudinal regression analyses. The cognitive function was represented as a global cognitive score derived from converting the raw scores of 19 cognitive tests to *Z* scores and then averaging them. The global cognitive score at the last visit pre‐death was used for cross‐sectional analyses. The longitudinal cognitive trajectories were derived from linear mixed‐effects models estimating individual annual rates of cognitive change. Covariates included age at death, sex, *post mortem* interval (PMI), and interval between last visit and death. For longitudinal cognitive function models, time interval in years between a visit with a cognitive exam and the last visit was also added as a covariate. Statistical analyses were performed using R (version 4.2.1). Multiple testing was controlled using FDR correction per tissue‐and‐outcome combination across all genes tested.

### 2.5 Gene expression in ROSMAP single‐nucleus RNAseq and AD endophenotypes

We further investigated cell type–specific gene expression profiles linked to AD endophenotypes for the genes with causal evidence from image‐directed analyses, using the single‐nucleus RNAseq (snRNAseq) data derived from DLPFC brain specimens of 424 post‐QC ROSMAP participants (syn31512863).^31^ Eight major cell types, CUX2+/CUX2− excitatory neurons, inhibitory neurons, astrocytes, microglia, oligodendrocytes, oligodendrocyte precursor cells (OPCs), and endothelial cells, were included in the analyses. Briefly, exclusion criteria applied to genes with expression in < 10% of all cells and to cells that had > 20,000 or < 200 total RNA unique molecular identifiers (UMIs), or with > 5% mitochondrial‐mapped reads. The gene count matrix was derived from UMI count data from the RNA assay and normalized and scaled using the “sctransform” R package https://github.com/satijalab/sctransform). The NEBULA‐HL method, implemented within the NEBULA R package (version 1.2.0),^32^ was used to fit negative binomial lognormal mixed models in a cell type–specific manner on the snRNAseq data.^33^ Differential gene expression was assessed between participants with normal cognition (“cogdx” = 1, *N* = 142) and those with AD dementia (“cogdx” = 4 or 5, *N* = 157), adjusting for age at death, sex, and PMI. These same covariates were included in modeling the associations between snRNAseq profile of genes and AD endophenotypes, including Aβ load (IHC staining), neurofibrillary tangle density (IHC staining), and both cross‐sectional and longitudinal measures of cognitive function. Statistical analyses were performed using R (version 4.2.1). Multiple testing was controlled using the FDR correction procedure per cell type within each tissue and outcome pair across genes tested.

### 2.6 Image‐agnostic analysis

For the image‐agnostic analysis, we used the NeuroimaGene^20^ resource to examine associations between the 23 previously described AD TWAS genes and cortical and subcortical NIDP morphology as generated by the FIRST and FAST image segmentation protocols.^34^, ^35^, ^36^ We applied an atlas‐wide FDR of 0.05 as the significance threshold. Next, we implemented the MR joint tissue imputation (JTI) pipeline to perform post hoc causal inference on these associations.^28^

### 2.7 Tissue context validation in the Human Protein Atlas

The Human Protein Atlas (HPA) provides bulk RNAseq data across 253 different tissues, encompassing 19,023 genes.^37^ We accessed the data release that includes only information from HPA study participants instead of the consensus data to avoid sample overlap with GTEx. To validate the tissue contexts in which the AD–GReX associations were identified from the image‐agnostic analysis, we first selected the genes from the association analysis on the full catalog of NIDPs that demonstrated significant causal effects on both AD and neuroanatomical measures in MR. We then identified the tissue contexts in which these associations reached significance.^28^ We next mapped these tissues to those included in the HPA according to their neuroanatomic identifiers. For each gene, we extracted the measured expression across all 253 tissues from the HPA. We scored tissues on a sliding scale from 0 (lowest measured gene expression) to 1 (highest). We then ranked each tissue–gene pair according to the score of the tissue.

### 2.8 Genetic covariance between family history of dementia and structural NIDPs

We identified loci associated with parental dementia from a GWAS by Marioni et al., which leveraged self‐reported phenotype data from > 300,000 individuals from the UKB.^5^ As noted by Marioni et al., the parental dementia variable was captured through a questionnaire asking if either biological parent had “Alzheimer's disease/dementia,” and thus may not distinguish AD from other dementia subtypes. As such, we refer to the phenotype in this study as “parental dementia” or “family history of dementia” recognizing that results are not specific to AD. We applied GeNetic cOVariance Analyzer (GNOVA) to perform genetic covariance analysis between the Desikan atlas NIDPs in the UKB and the questionnaire‐based parental dementia status using summary statistics.^38^ GNOVA is robust to potential sample overlap across GWAS datasets. Linkage disequilibrium (LD) scores were derived from the subset of European ancestry reference data from the 1000 Genomes Project.^39^ We retained NIDPs with covariance measures for which *P*_FDR_ < 0.05.

### 2.9 Genetic covariance of AD status and structural NIDPs

We identified loci associated with clinical/pathology‐confirmed AD from the GWAS by Kunkle et al. (*N*_cases_ = 35,274, *N*_controls_ = 59,163).^4^ We then performed genetic covariance analyses between 490 structural NIDPs, including those from the Desikan, Desikan–Killiany–Tourville (DKT), and Fischl subcortical atlas, and AD status using GNOVA. Significance was defined as *P*_FDR_ < 0.05. Additionally, we applied GNOVA to test for the genetic covariance between AD^4^ status and 46 hippocampal NIDPs, applying the same significance threshold as above.

### 2.10 Local genetic covariance of dementia family history and structural NIDPs

We investigated the local genetic covariance between parental AD status (using the Marioni et al.^5^ GWAS) and each previously implicated structural NIDP associated with GReX of previously implicated genes, using the SUPER GeNetic cOVariance Analyzer (SUPERGNOVA) tool.^40^ SUPERGNOVA leverages LDetect to partition the genome into local LD blocks for examining local genetic covariance for each GWAS pair.^41^ Significance was defined as the nominal SUPERGNOVA *P* < 0.05. In addition, we required the nominally significant genetic covariance signals to fall within two megabases upstream and downstream of the corresponding gene (based on genome build 37 coordinates).

### 2.11 Phenotypic correlation between NIDPs and family history of dementia

We assessed the significance of empirical measurement differences in NIDPs across UKB individuals with and without a family history of dementia. We first identified individuals with a family history of dementia according to the disease code 29626 (AD) recorded in either of the two data fields, “illnesses of father” and “illnesses of mother.” Each individual was also tagged with data fields for sex, age at recruitment, and AD diagnosis to be used as covariates and filtering criteria. Among individuals who self‐reported either or both of their parents with AD, we excluded those with neurologic diagnoses according to the presence of “G” category International Classification of Diseases (ICD) codes, as well as individuals with cerebrovascular events as noted by the “I6” prefix to ICD codes. Given the use of sex as a covariate, we excluded those individuals whose biological sex demonstrated a discrepancy with their self‐reported sex.

We first fit the NIDP data to a Weibull distribution using a Cullen and Frey chart. We then regressed the NIDP against family history of dementia with sex and age at recruitment as covariates. Using the Generalized Additive Models for Location Scale and Shape package in R, we performed this regression against a Weibull distribution: NIDP ∼ ADfamHx * Sex * `Age at recruitment`.^42^ NIDPs with distributions that did not match the Weibull distribution were instead fit assuming a normal distribution. Nominally significant association of dementia family history with a NIDP was defined as *P* < 0.05. We repeated this analysis for the subset of NIDPs that demonstrated nominally significant correlations with dementia family history.

## 3 RESULTS

### 3.1 Clinically informed image‐directed association testing of AD‐associated genes

This study assessed the relationship between genetic risk of AD and neuroimaging‐derived features by leveraging multiple data sources (Figure 1A, Table S1). In this first analysis, we examined whether the GReX of AD‐associated genes exhibits statistically significant associations with brain measures, or NIDPs, previously implicated in clinical AD studies. We selected 23 candidate genes identified in our prior AD TWAS study on large‐scale ADGC/IGAP consortium data^3^ (*N*_cases_ = 20,613, *N*_controls_ = 37,658, Table S2 in supporting information), with causal inference validated. We then proceeded with this hypothesis‐driven analysis by using NeuroimaGene (Figure 1B).^20^ The NIDPs were selected based on clinical relevance as reported by Westman et al.’s study^24^ (Methods, Figure 1E, Table S3 in supporting information), which used consistent preprocessing, parcellation, and segmentation approaches consistent with UKB, ensuring methodological comparability and rigor.

Statistical significance was defined as a Benjamini–Hochberg FDR threshold of 0.05 (Table S4 in supporting information). We then performed causal inference on these GReX–NIDP associations using the MR JTI methodology.^28^ Our assumption of causality depends on having genetic instruments (eQTLs) that are (1) strongly associated with GReX, (2) independent of confounders affecting both GReX and NIDPs, and (3) influence NIDPs only through GReX (Table S5 in supporting information).^28^

### 3.2 *PSMC3* is an AD‐associated gene that affects AD‐associated NIDPs

**FIGURE 2:**
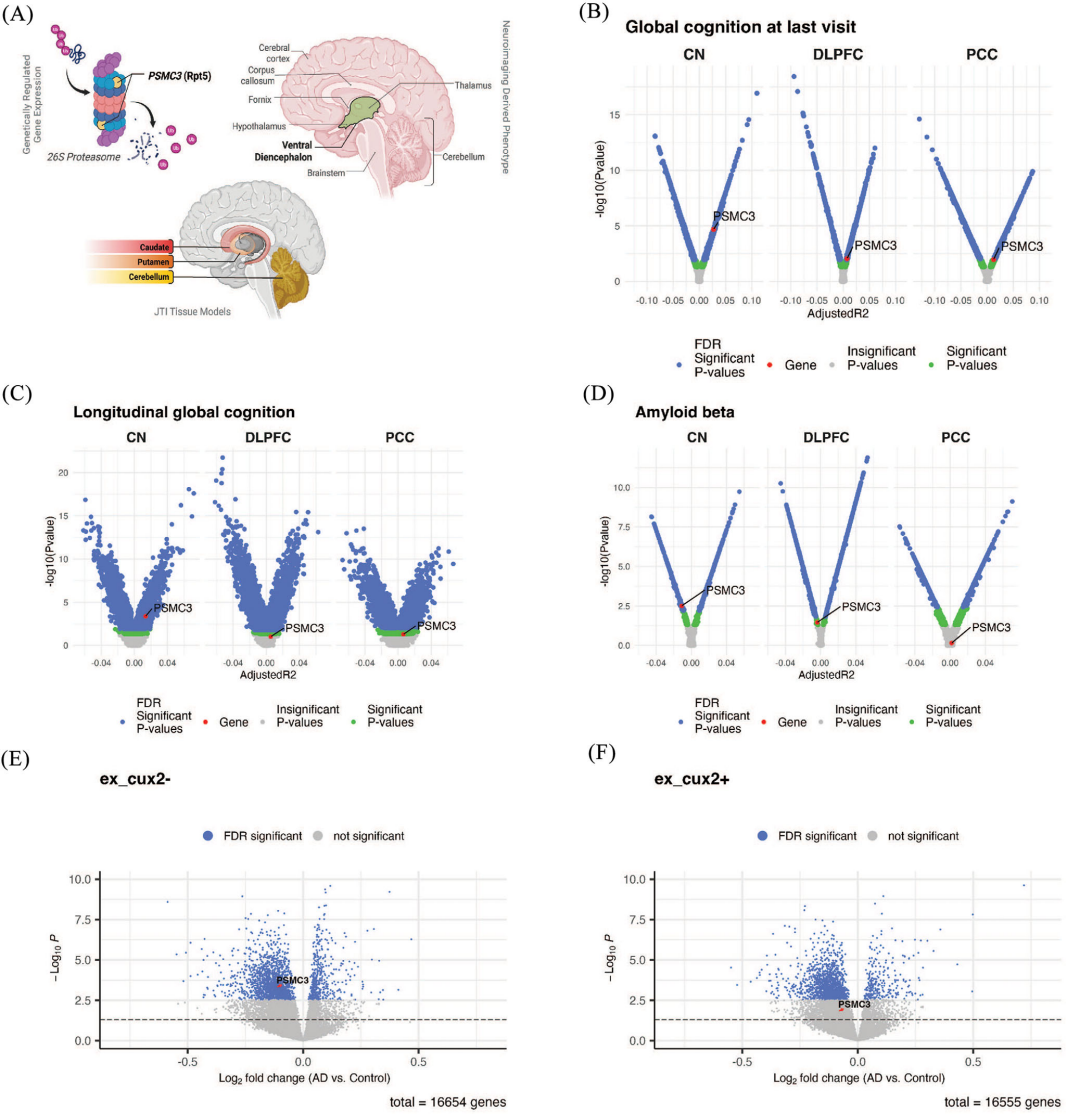
Visual summary of MR findings for *PSMC3*. A, GReX of *PSMC3* is associated with the volume of the ventral diencephalon according to three different gene expression models trained in the caudate, putamen, and cerebellum. *PSMC3* encodes the protein Rpt5, which acts as an ATPase involved in substrate translocation in the 26S proteasome complex. *PSMC3* expression levels from bulk RNAseq associated with (B) global cognition score at last visit, (C) longitudinal global cognition trajectory, and (D) amyloid beta deposition. The *x* axis in the volcano plot represents the adjusted *R* ^2^ value derived from the regression analyses. *PSMC3* expression levels from snRNAseq associated with AD case/control status in (E) CUX2− and (F) CUX2+ excitatory neurons. The *x* axis represents the log‐fold change of *PSMC3* expression between AD cases and normal controls. The *y* axis in (B–F) represents the −log_10_ *P* values. The blue dots represent significant *P* values (*P*_FDR_ < 0.05), the green dots represent nominally significant *P* values, and the gray dots represent non‐significant *P* values. The red dots and arrows highlight *PSMC3*. AD, Alzheimer's disease; CN, caudate nucleus; DLPFC, dorsolateral prefrontal cortex; FDR, false discovery rate; GreX, genetically regulated gene expression; JTI, joint tissue imputation; MR, Mendelian randomization; PCC, posterior cingulate cortex; RNAseq, RNA sequencing; snRNAseq, single‐nucleus RNA sequencing.

GReX for 7 of the 23 candidate genes showed associations with NIDPs (Table S4). The most common NIDPs implicated by AD genes included the left ventral diencephalon volume and the right frontal pole thickness. The most significant association of this image‐directed analysis was between *PSMC3* and the volume of the left ventral diencephalon (*P*_FDR_ < 0.05). Additionally, post hoc MR JTI analysis indicated a causal effect of *PSMC3* GReX on the volume of the ventral diencephalon, with supporting evidence from tissue models derived from the caudate, putamen, and cerebellum (Table S5). The volume of the ventral diencephalon has been independently noted to differ in AD patients compared to healthy controls.^24^ The *PSMC3* gene encodes the ATPase 3 protein for the 26S proteasome involved in the digestion of proteins tagged for cellular clearance. *PSMC3* enables the full proteasome complex to cleave ubiquitinated peptides in an ATP‐dependent manner (Figure 2A). The ventral diencephalon, associated with *PSMC3* and implicated in both AD and AD neuroimaging, involves multiple subcortical structures, including the hypothalamus, mammillary body, subthalamic nuclei, substantia nigra, red nucleus, lateral geniculate nucleus (LGN), and medial geniculate nucleus (MGN).

### 3.3 Transcriptome analysis implicates *PSMC3* in AD endophenotypes

To further investigate the relationship between *PSMC3* gene expression and AD pathophysiology, we performed several tests using bulk and snRNAseq data from ROSMAP,^30^, ^31^ a deeply phenotyped aging and AD cohort with extensive multi‐omics data (see Methods section). In bulk RNAseq analyses of the DLPFC, PCC, and CN, we identified three significant associations (*P*_FDR _< 0.05) between *PSMC3* expression and AD endophenotypes. These include global cognition score at last visit (*β* = 0.789, *P*_FDR _= 4.91 × 10^−4^, Figure 2B), longitudinal trajectory of global cognition (*β* = 0.062, *P*_FDR _= 0.00390, Figure 2C), and Aβ depositions (*β* = –0.550, *P*_FDR _= 0.0324, Figure 2D) in the CN tissue. An additional significant association between *PSMC3* expression and global cognition at the last visit (*β* = 0.513, *P*_FDR _= 0.0416, Figure 2B) was observed in the DLPFC tissue. All bulk RNAseq results and additional visualizations are presented in Table S6 and Figure S2 in supporting information. In the snRNAseq analyses of DLPFC, we observed a significant association between *PSMC3* expression in CUX2− excitatory neurons and AD dementia status (log fold change [logFC] = –0.114, *P*_FDR _= 0.019, Figure 2E), yet not in CUX2+ excitatory neurons (logFC = –0.082, *P*_FDR _= 0.123, Figure 2F). All snRNAseq results and additional visualizations are available in Table S7 and Figures S3–S7 in supporting information.

### 3.4 Atlas‐wide association testing of AD‐associated genes and cortical NIDPs

**FIGURE 3:**
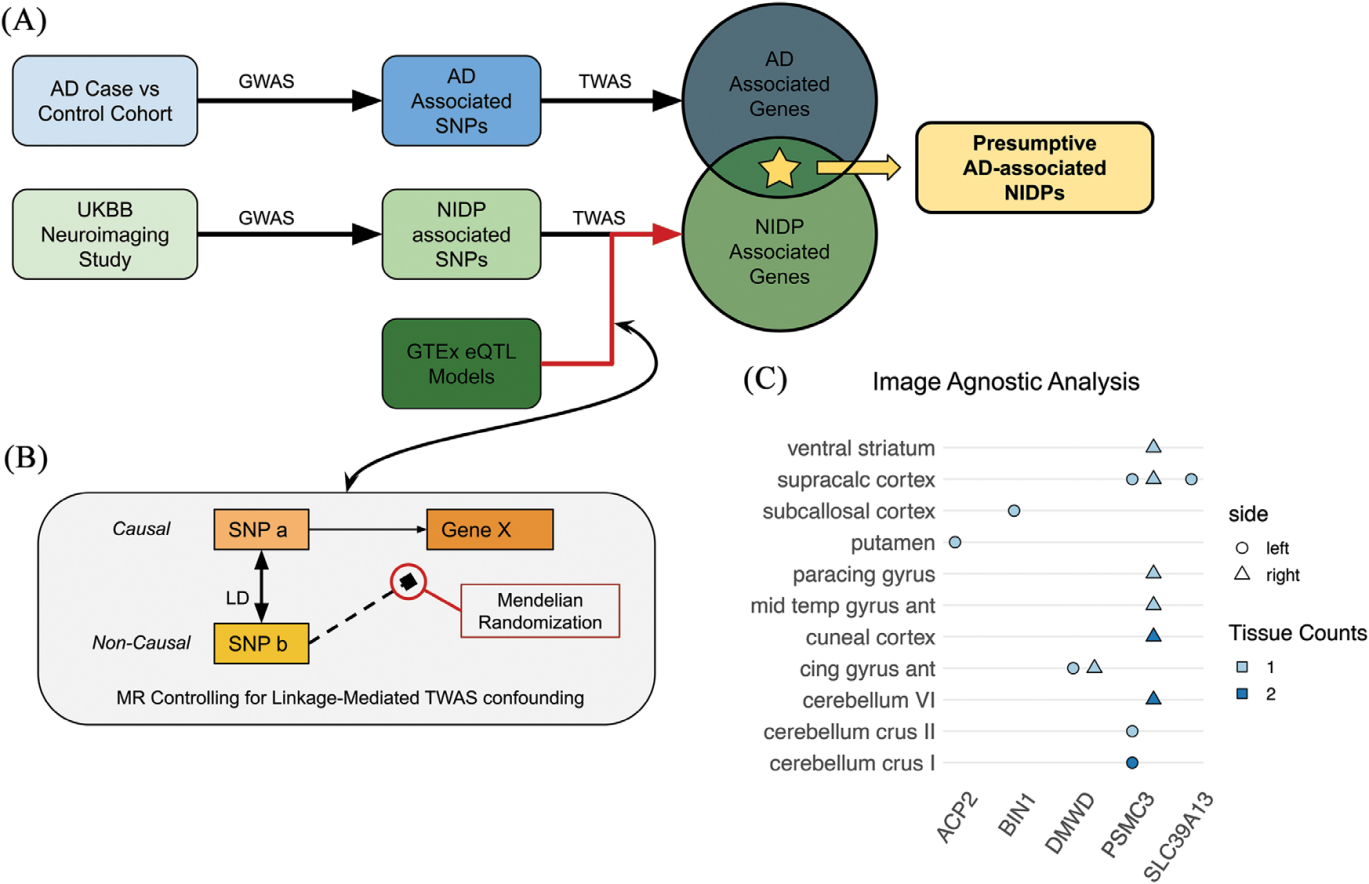
Summary of image‐agnostic workflow and principal findings. A, Schematic overview of TWAS‐based intersectional methodology for the image‐agnostic approach. B, Conceptual illustration of horizontal pleiotropy mediated by linkage disequilibrium and the application of MR to mitigate pleiotropic bias. C, NeuroimaGene associations resulting from the image‐agnostic analysis. Data points represent statistically significant associations between AD‐associated eGenes (*x* axis) and NIDPs corresponding to cortical and subcortical parcellations of the brain (*y* axis). The color of each tile represents the number of tissue‐specific gene expression models in which the eGene–neuroimaging association reached statistical significance in the MR JTI causal inference analysis. The shape of the point specifies the lateral localization of the region to either the left or right hemisphere. expression quantitative trait loci, expression quantitative trait loci; GTEx, Genotype–Tissue Expression project; GWAS, genome‐wide association study; JTI, joint tissue imputation; MR, Mendelian randomization; NIDP, neuroimaging‐derived phenotype; SNP, single‐nucleotide polymorphism; TWAS, transcriptome‐wide association study; UKBB, UK Biobank.

After identifying associations between known AD genes and brain regions previously implicated in AD, we next performed an atlas‐wide association analysis via NeuroimaGene (Figure 1B) to assess associations between AD genes and all structural brain measures, extending our investigation beyond hypothesis‐driven assessment constrained by clinical neuroimaging priors. We tested all T1‐derived cortical and subcortical NIDPs from the FIRST^36^ and FAST^35^ cortical and subcortical segmentation protocols for associations with the 23 AD genes from the previously described study (Table S8 in supporting information). For significant GReX–NIDP associations (*P*_FDR _< 0.05), we tested for causal effects using MR JTI as before.^28^ This approach identified five AD‐associated genes, *ACP2*, *BIN1*, *DMWD*, *SLC39A13*, and *PSMC3*, with putatively causal effects on 13 different NIDPs (Figure 3 and Table S9 in supporting information). Again, *PSMC3* showed the most GReX–NIDP associations, appearing significant in six tissues (*P*_FDR _< 0.05) and implicating eight different NIDPs. The GReX–NIDP associations for the remaining genes with causal evidence, *ACP2*, *BIN1*, *DMWD*, and *SLC39A13*, were each significant in a single tissue (Table S9 and Figure S8 in supporting information).

### 3.5 Gene expression validation in the HPA

The NeuroimaGene atlas‐wide, discovery‐oriented analysis aimed to identify previously unrecognized associations between GReX of AD‐associated genes and NIDPs without relying on prior clinical neuroimaging knowledge. Given that the TWAS framework implemented in NeuroimaGene assesses GReX–NIDP associations in tissue‐relevant contexts, validating the biological relevance of tissue‐specific gene associations is critical. Therefore, we sought to further evaluate the biological plausibility of our findings by examining the target gene expression levels in the corresponding tissues where it is predicted to influence brain structural measures, using data from the HPA^37^ resource. HPA provides bulk RNAseq data collected across 253 different tissues, spanning 19,023 genes. Of the seven tissues represented in MR significant gene–tissue associations from our atlas‐wide image‐agnostic analysis, six were available in the HPA (Table S10 in supporting information). Four of the five genes from these MR significant associations showed evidence of causality in a single tissue. All four of these genes were expressed in the corresponding tissue in the HPA (Figures S9 and S10 in supporting information).

To evaluate the tissue specificity of gene expression across all available tissues, we systematically ranked all 253 tissues based on their measured expression levels (Methods). Notably, *PSMC3* was observed to be expressed in the HPA in the same five discovery tissues (Figure S11 in supporting information). The *PSMC3* expression in the caudate nucleus was high, ranking in the 95th percentile among all 253 tissues in the HPA. This increase approaches statistical significance (*P* = 0.051). This observation aligns with the *PSMC3* GReX associations identified through image‐directed analyses in the caudate, putamen, and cerebellum, with the volume of the ventral diencephalon (Table S5). Overall, we showed that the tissue contexts in which the GReX of AD‐associated genes are predicted to have an effect are plausible according to expression data from a second, independent cohort.

### 3.6 Genetic covariance between family history of dementia and structural NIDPs

While TWAS analyses examine the subset of genetic variation that manifests as alterations in the expression of specific RNA transcripts, gene expression changes represent only one facet of the genetic risk underlying AD. To more comprehensively evaluate a broader range of the genetic risk factors for AD, we complemented our TWAS framework with genetic covariance approaches (Figure 1C). First, we investigated the neurological implications of SNP‐mediated dementia risk among individuals with a family history of dementia. To exclude the broad array of dementia risk factors transmitted through non‐genetic mechanisms, we calculated the genetic covariance between familial dementia history and various NIDPs. We hypothesized that this approach would identify NIDPs associated with AD in clinical neuroimaging studies.

**FIGURE 4:**
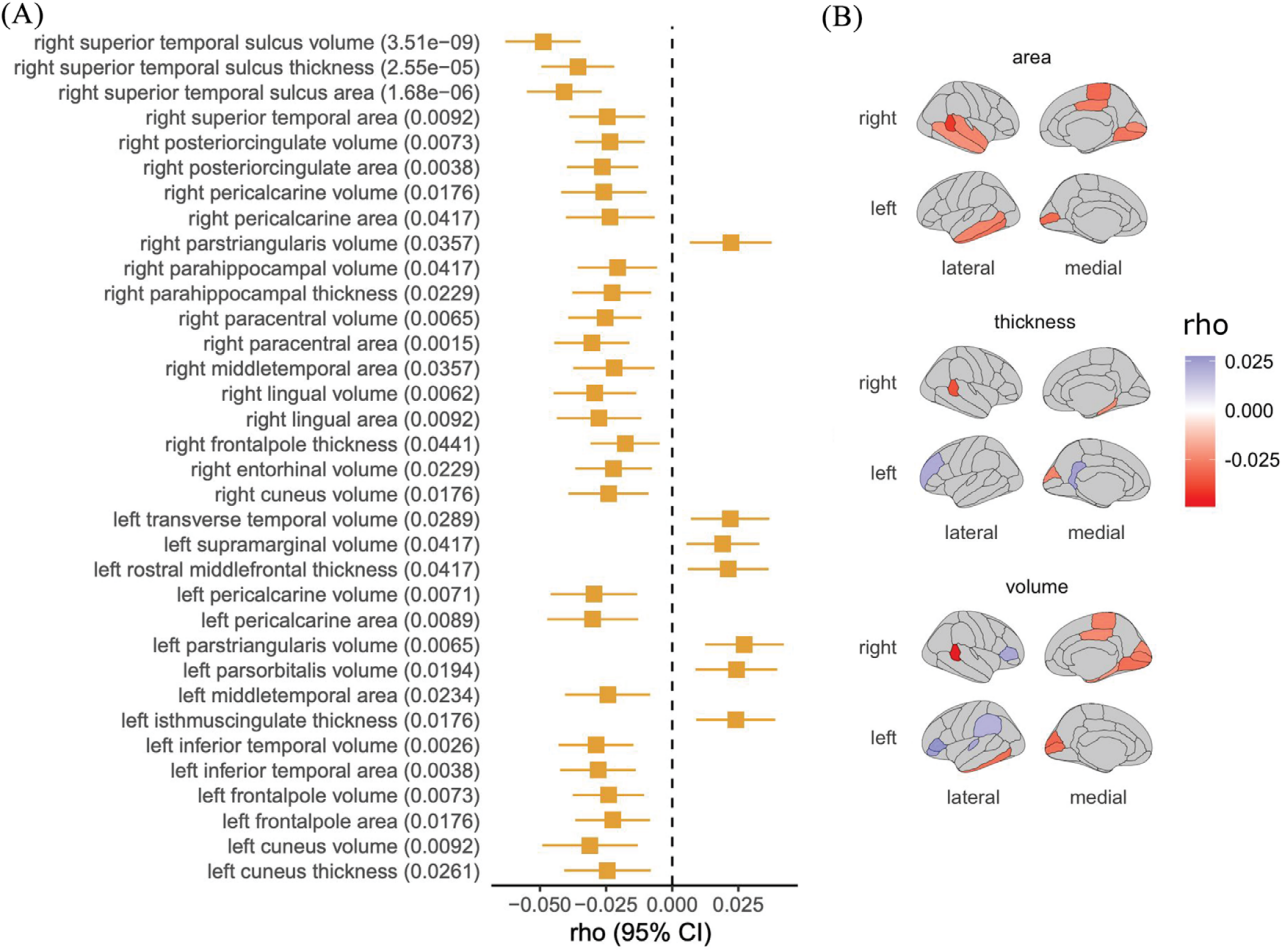
Bonferroni significant global genetic covariance between NIDPs and family history of dementia. A, Genetic covariance was calculated using GNOVA. Error bars represent standard errors. B, Two‐dimensional visualization of FDR‐significant NIDPs with genetic architecture that covaries with dementia family history. CI, confidence interval; FDR, false discovery rate; GNOVA, GeNetic cOVariance Analyzer; GWAS, genome‐wide association study; NIDP, neuroimaging‐derived phenotype.

Parental dementia status was used as a proxy for genetic risk. Genetic covariance analyses were performed with GNOVA using 202 genome‐wide GWAS summary statistics of structural NIDPs from the UKB and one publicly available GWAS summary statistics for family history of dementia.^5^ Thirty‐four NIDPs demonstrated significant (*P*_FDR_ < 0.05) genetic covariance with family history of dementia (Figure 4A,B and Table S11 and Figure S12 in supporting information). These neuroimaging‐derived traits encompassed measures of surface area, volume, or cortical thickness across different regions within the left or right hemisphere. Several of these cortical regions have been previously associated with prodromal AD according to Braak staging of both Aβ and tau progression.^43^ Multiple regions were implicated in both the image‐agnostic and genetic covariance analyses, including the calcarine region, cingulate gyrus, middle temporal gyrus, and cuneus.

### 3.7 Genetic covariance of AD status and structural NIDPs

**FIGURE 5:**
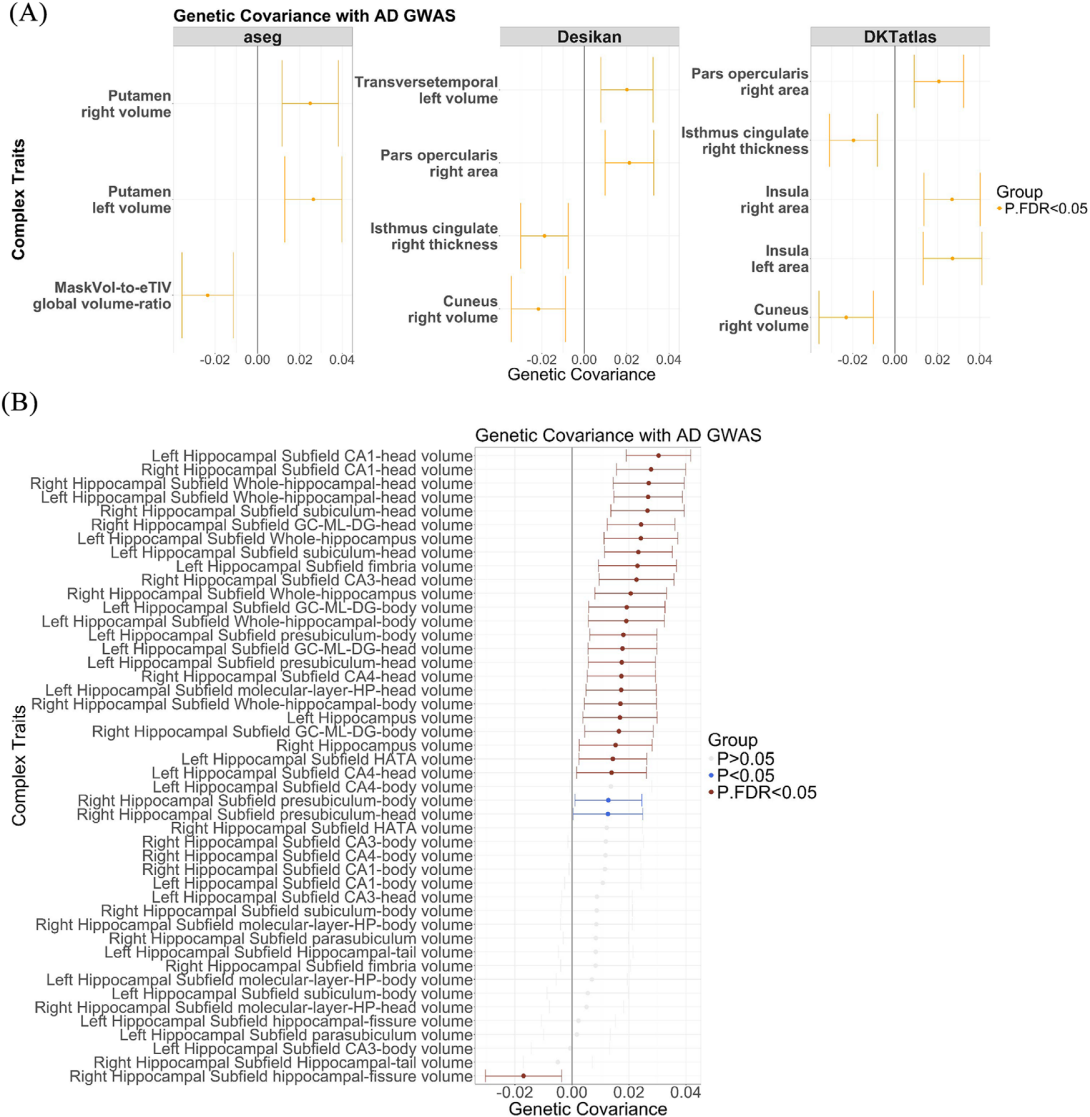
Genetic covariance between NIDPs and clinical AD risk. A, Genetic covariance calculated using GNOVA between NIDPs, processed using aseg, Desikan, and DKT atlases, and clinical AD status from Kunkle et al. The *x* axis depicts the estimates of genetic covariance. The *y* axis lists the NIDPs. Each facet displays results from a different atlas. B, The genetic covariance results between hippocampus NIDPs and clinical AD status from Kunkle et al. The *x* axis depicts the genetic covariance estimates, while the *y* axis displays the NIDPs. The significant results are shown in dark red, nominally significant results in light blue, and non‐significant results in gray. AD, Alzheimer's disease; DKT, Desikan–Killiany–Tourville; GNOVA, GeNetic cOVariance Analyzer; GWAS, genome‐wide association study; NIDP, neuroimaging‐derived phenotype.

Next, we performed a more phenotypically specific analysis to evaluate the shared genetic architecture between a clinical AD diagnosis and NIDPs, using the GNOVA^38^ methodology (Figure 1C). In this analysis, we targeted structural NIDPs processed using Fischl subcortical, Desikan, and DKT atlases, as well as the NIDPs measured from the hippocampus. We identified significant (*P*_FDR_ < 0.05) genetic covariance between AD diagnosis status and the volume of the cuneus, the thickness of the cingulate gyrus (isthmus), and the surface area of the right pars opercularis across the Desikan and DKT atlases. Additionally, we identified significant genetic covariance (*P*_FDR_ < 0.05) in the right and left surface area of the insula from the DKT atlas and the volume of the left transverse temporal gyrus from the Desikan atlas. The volume of the putamen on both hemispheres reached statistical significance (Figure 5A and Table S12 and Figure S13 in supporting information).

Regarding the hippocampal findings, 25 of the 46 subfields (54%) demonstrated significant (*P*_FDR_ < 0.05) genetic covariance with AD status (Figure 5B and Table S13 in supporting information). The subcortical features with the greatest genetic covariance measures include the bilateral heads of cornu ammonis (CA) region 1, bilateral measures of the whole hippocampal head, and the volume of the right head of the subiculum.

### 3.8 Local genetic covariance of parental dementia and structural NIDPs

Although global genetic covariance captures genome‐wide shared genetic architecture between two traits, it does not delineate localized regional variation. By leveraging SUPERGNOVA, we examined the potential for convergence between our prior gene‐based image‐directed analysis and local genetic covariance analysis. We identified hundreds of regions across the genome with a statistically significant (*P* < 0.05) local genetic covariance between structural NIDPs linked to AD‐associated genes from our image‐directed analysis (Table S4) and the genetic architecture of family history of dementia (Figures S14–S22 in supporting information). Each of these regions represents a genomic hotspot at which a greater degree of similarity exists than would be expected by chance between the genetic underpinning of an NIDP and that of parental dementia.

We next assessed whether any significant NIDP‐parental dementia covariance loci lie within 2 Mb of an AD‐associated gene linked to the respective NIDP. Notably, we found such a locus on chromosome 11 within 2Mb of the *MS4A4E* region. The *MS4A4E* region demonstrated a positive genetic covariance with two NIDPs: thickness measures of the right pars opercularis and the right isthmus of the cingulate cortex, as captured by the Desikan atlas (Table S14 in supporting information). GReX of *MS4A4E* was also associated with the morphology of these same two regions in the image‐directed analysis (Table S4). *MS4A4E* is a membrane‐spanning protein that has been previously associated with AD and cerebral amyloid angiopathy, further supporting the biological relevance of this locus.^44^, ^45^

### 3.9 Phenotypic correlation between NIDPs and family history of dementia

**FIGURE 6:**
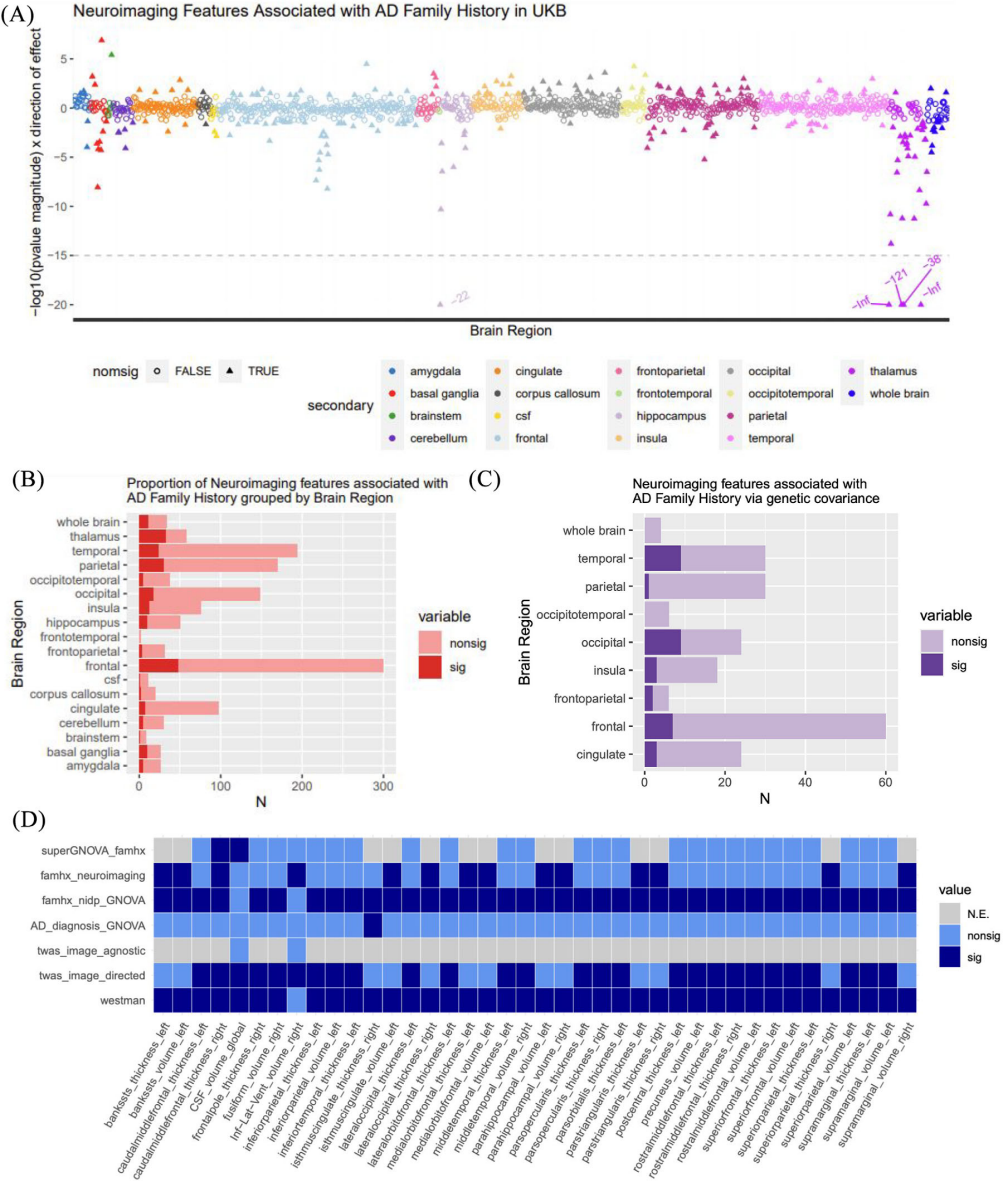
Summary of associations between NIDPs and AD endophenotypes. A, Neuroimaging features associated with family history of dementia. The *x* axis represents the surface area, volume, and thickness of cortical and subcortical regions as determined by the UKB. The *y* axis represents the negative log of the effect size magnitude. This measure reflects the association between family history of dementia and the measurement of the region, adjusted for sex and age. Nominally significant findings (*P* < 0.05) are solid triangles while non‐significant associations are empty circles. Panels separate findings according to effect size and direction of effect. Points are colored according to the neuroimaging atlas used to parcellate the brain regions in MRI protocols. B, We categorized each NIDP according to the named brain region (*y* axis). The *x* axis represents the number of NIDPs associated with parental dementia history. The solid red represents the nominally significant associations, while the pale red represents non‐significant associations. C, Subset of NIDPs from (B) with an FDR‐significant global genetic covariance with parental dementia. The NIDPs with both an FDR‐significant genetic covariance and nominally significant phenotypic correlation are shown in dark purple. The NIDPs with an FDR‐significant genetic covariance but non‐significant phenotypic correlation with parental dementia are shown in light purple. D, Tile plot of neuroimaging features (*x* axis) with nominally significant associations with at least two AD‐related variables (*y* axis). Significant NIDP‐phenotype associations are colored deep blue, while non‐significant associations are light blue. Those associations that were not evaluated (N.E.) are shaded gray. AD, Alzheimer's disease; FDR, false discovery rate; GNOVA, GeNetic cOVariance Analyzer; MRI, magnetic resonance imaging; NIDP, neuroimaging‐derived phenotype; UKB, UK Biobank.

Having examined the genetic overlap between NIDPs and family history of dementia, we next investigated whether such genetic similarity translated into observable phenotypic differences in neuromorphology among individuals stratified by their family history of dementia status (Figure 1D and Figure S23 in supporting information). The family history of dementia status information for this phenotypic analysis was defined using UKB data fields and ICD codes (Methods, Tables S15 and S16 in supporting information). We used multivariate linear regression to examine the relationship between family history of dementia and T1 structural NIDPs (Figure 6A and Table S17 in supporting information). We identified 229 NIDPs with a nominally significant association (*P*_uncorrected_ < 0.05) with dementia family history (Figure 6B), of which 31 surpassed the Bonferroni significance threshold. Of the 31 Bonferroni‐significant associations, the majority involved the thalamus, followed by the frontal cortex, the hippocampus, and the basal ganglia. Overall, 94% of these structural measures demonstrated changes consistent with atrophy (reductions in volume or increases in surface area; Table S17). Both phenotypic and global genetic covariance analyses between family history of dementia and NIDPs revealed enrichment of significant associations in frontal, temporal, and occipital regions (Figure 6C and Table S18 in supporting information). Across all analyses (Figure 1F), the most consistently implicated feature was the thickness of the right caudal middle frontal gyrus (Figure 6D and Table S18).

## 4 DISCUSSION

Several studies have sought to identify neuroimaging‐derived and gene expression correlates of AD.^3^, ^10^, ^46^, ^47^, ^48^ Here, we performed analyses leveraging large extant resources (Figure 1A and Table S1) to integrate multiple layers of AD genetic risk, including GReX of AD‐associated genes (Table S2), genome‐wide summary statistics for family history of dementia and clinical AD diagnosis, and genetic predictors of NIDPs, with the goal of extending the mechanistic and functional understanding of AD risk (Figure 1F). We presented substantial evidence linking the expression of known AD risk genes with the morphology of specific brain regions known to be altered in patients with the disease. Genome‐wide and local genetic covariance results showed that either a family history of dementia or a disease diagnosis is associated with detectable changes in neuromorphology measurable via MRI.

Among the AD‐related genes, *PSMC3* showed notable links with AD endophenotypes. The crucial role of *PSMC3* in effective intracellular protein degradation is of particular interest given the role of aberrant protein aggregates in AD pathophysiology. Our MR analyses support a potential causal connection between *PSMC3* expression and multiple quantitative measures of brain morphology in healthy patients, identified through both image‐directed and image‐agnostic methods (Tables S5 and S9). Results from the ROSMAP bulk RNAseq data identified significant positive associations between *PSMC3* expression and overall cognition at both cross‐sectional timepoints and longitudinal trajectories, implying that reduced *PSMC3* levels in the CN and DLPFC tissues correlate with worse cognitive performance (Table S6). Additionally, a negative association was found between *PSMC3* expression and Aβ accumulation in CN, implying that increased *PSMC3* expression may confer protection against Aβ buildup (Table S6). snRNAseq data from ROSMAP identified a significant reduction of *PSMC3* expression in AD dementia compared to cognitively normal participants, specifically in CUX2− excitatory neurons of the DLPFC, indicating dysregulation of *PSMC3* in AD dementia patients (Table S7).

An additional notable finding is the significant causal association (from the MR analysis) between *PSMC3* in the caudate tissue and the volume of ventral diencephalon (DC), which is noted to be different in AD patients versus normal controls (Table S5).^24^, ^49^, ^50^ Coupled with evidence of protective effects against Aβ deposition and positive cognitive outcomes observed via its gene expression in the caudate nucleus of ROSMAP participants, this provides additional supporting evidence for *PSMC3*’s role in AD risk. Overall, our approach highlights how linking *PSMC3* GReX with NIDPs may inform hypotheses regarding brain regions influenced by *PSMC3* expression in the context of AD.

While *PSMC3* emerges as a robust candidate supported by both image‐directed and image‐agnostic NeuroimaGene TWAS analyses, the image‐agnostic analysis with MR causal inference further implicates *DMWD*, *BIN1*, *SLC39A13*, and *ACP2* as additional candidate genes. *DMWD*  is particularly notable: our data provide evidence that genetically regulated expression of this gene is associated with both AD risk and altered morphology of the bilateral anterior cingulate gyrus. A prior publication provides corroborating evidence. Ghanbari et al. demonstrate that a variant disrupting a microRNA binding site in *DMWD* is associated with AD, providing mechanistic support for the relevance of *DMWD* expression regulation in AD.^51^

Synthesizing the neuroimaging findings across the genetic, transcriptomic, and clinical analyses, the top NIDPs implicated several regions with previous evidence for roles in AD (Figure 6D and Table S18). The increase of cerebrospinal fluid (CSF) volume in response to global cortical atrophy is one of our top findings and a well‐documented association with AD.^52^ The NIDP with the most consistent evidence for association with AD‐related genetic risk is the right middle caudal frontal gyrus. Atrophy of this region has been robustly associated with AD.^53^ Additional outside work highlights the atrophy of the right inferior temporal cortex as causal in AD, a finding which we replicated in genetic covariance analyses of AD status.^10^ Our fourth finding highlights the thickness of the isthmus in the right cingulate gyrus. Two recent publications have linked the white matter architecture of the cingulate bundle to memory and cognitive performance as well as AD risk genes and AD polygenic risk scores, reinforcing its relevance in AD pathophysiology.^54^, ^55^

Of note, we recapitulated several previous findings of atrophy in the hippocampi.^56^, ^57^ Zammit et al.^56^ examined the relationship between hippocampal subfield morphology and cognitive performance as measured through figure and verbal memory testing in healthy adults. They identified statistically significant associations between volume of CA1, the subiculum, and the whole hippocampus and cognitive measures. Additional studies have linked the subiculum and CA1 to AD.^58^, ^59^, ^60^ The subiculum, CA1, and the whole hippocampus subregions in both hemispheres constitute six target subregions for our analysis. All six occur within the top seven results of our genetic covariance analysis, ranked by either *P* value or covariance (Figure 5B and Table S13). Hippocampal atrophy is an early finding in AD and it has been hypothesized that these changes may reflect the region's increased vulnerability to toxins such as Aβ.^61^ Together, these data suggest that the genetic architecture of these hippocampal subregions itself is associated with AD and, relying on Zammit et al., that the specific subregions are involved in verbal and visual recall.

A strength of our omics‐informed neuroimaging analyses is that they evaluate drivers of neurologic morphology at the gene expression level rather than focusing on individual SNPs. The interpretation of gene expression is generalizable across populations in a way that SNP variation is not. Because we performed the neuroimaging TWAS analyses in a large population that is largely free of overt AD, our results reveal endogenous associations between gene expression and neuromorphology. This mitigates imaging‐based confounding introduced by aging and disease‐mediated brain changes.^22^ The liability model of disease holds that risk is manifested through an accumulation of factors that drive a patient toward the upper end of a risk distribution.^62^ These same risk factors are distributed broadly across the population, usually at insufficient doses to produce overt symptoms. The data presented here suggest that genetic and transcriptomic risk factors for AD result in subclinical biochemical and biological consequences that can be observed outside of the context of overt disease, evidence that could be missed in studies restricted to disease‐affected cohorts. Through this multidisciplinary approach, we present a network of interrelated AD endophenotypes, linking genetic, transcriptomic, and neuroimaging variables into a cohesive biological system (Figure 1F).

Nevertheless, despite the convergence of neuroimaging findings across genetic, transcriptomic, and epidemiologic risk factors with the clinical AD diagnosis, it is possible that over‐ or underexpression of gene products may have different consequences in the context of disease relative to states of health.

Several additional limitations merit discussion. Our analyses assessed only the effects mediated through gene expression. Future studies on downstream molecular traits (e.g., protein or metabolite) are warranted. Regarding data quality, the dementia family history was self‐reported. While current evidence supports using this measure as a rough proxy for AD risk,^63^, ^64^ the diagnostic accuracy is likely to be reduced relative to a clinically phenotyped cohort.

We encountered several unexpected findings. Although multiple NIDPs demonstrated genetic covariance with family history of dementia, these regions largely differed from those linked with AD through the mechanism of GReX. Genetic covariance, in contrast to eQTL‐based analysis, does not evaluate gene regulatory effects. We suspect that this discrepancy is primarily the result of parental dementia and positive AD status being different phenotypes. While the family history of dementia may predispose individuals to neuropathophysiological development, the regions impacted appear to differ from those influenced by the expression of AD‐associated genes.

In summary, analysis of genetically determined expression of AD‐associated risk genes offers insight into potential neurological mechanisms by which AD‐associated variation confers disease risk. We showed that expression of *PSMC3* is associated with AD endophenotypes, including NIDPs, cross‐sectional and longitudinal cognitive function, and Aβ burden. Notably, in non‐AD patients, GReX of *PSMC3* leads to similar neuroanatomical changes, including changes in the volume of ventral DC, as those observed in individuals with AD. Additionally, we demonstrated that family history of dementia and AD status are both associated with neuromorphology via genetic covariance, albeit different regions. Last, we presented the strongest composite support for endogenous genetic influences on the frontal cortex, the global CSF volume, and the isthmus of the cingulate gyrus by AD‐predisposing genetic variation. By integrating multiple layers of functional evidence, we provide a prioritization schema for identifying and contextualizing biochemical entities as areas of focus for future mechanistic studies.

## CONFLICT OF INTEREST STATEMENT

The authors declare no conflicts of interest. Author disclosures are available in the supporting information.

## CONSENT STATEMENT

All participants provided informed consent in their respective cohort studies.

## Supporting information



Supporting Information

Supporting Information

Supporting Information

## References

[1] Gatz M , Reynolds CA , Fratiglioni L , et al. Role of genes and environments for explaining Alzheimer disease. Arch Gen Psychiatry. 2006;63(2):168‐174. doi:10.1001/archpsyc.63.2.168 16461860

[2] Bergem ALM , Engedal K , Kringlen E . The role of heredity in late‐onset Alzheimer disease and vascular dementia: a twin study. Arch Gen Psychiatry. 1997;54(3):264‐270. doi:10.1001/archpsyc.1997.01830150090013 9075467

[3] Chen HH , Petty LE , Sha J , et al. Genetically regulated expression in late‐onset Alzheimer's disease implicates risk genes within known and novel loci. Transl Psychiatry. 2021;11(1):1‐12. doi:10.1038/s41398-021-01677-0 34873149 PMC8648734

[4] Kunkle BW , Grenier‐Boley B , Sims R , et al. Genetic meta‐analysis of diagnosed Alzheimer's disease identifies new risk loci and implicates Aβ, tau, immunity and lipid processing. Nat Genet. 2019;51(3):414‐430. doi:10.1038/s41588-019-0358-2 30820047 PMC6463297

[5] Marioni RE , Harris SE , Zhang Q , et al. GWAS on family history of Alzheimer's disease. Transl Psychiatry. 2018;8(1):99. doi:10.1038/s41398-018-0150-6 29777097 PMC5959890

[6] De Deyn L , Sleegers K . The impact of rare genetic variants on Alzheimer disease. Nat Rev Neurol. 2025;21(3):127‐139. doi:10.1038/s41582-025-01062-1 39905212

[7] Liang Y , Nyasimi F , Melia O , et al. BrainXcan identifies brain features associated with behavioral and psychiatric traits using large‐scale genetic and imaging data. Dev Cogn Neurosci. 2025;73:101542. doi:10.1016/j.dcn.2025.101542 40101670 PMC11964658

[8] Li Y , Nan B , Zhu J . A structured brain‐wide and genome‐wide association study using ADNI PET images. Can J Stat Rev Can Stat. 2021;49(1):182‐202. doi:10.1002/cjs.11605 PMC8460073 34566241

[9] Xu Z , Wu C , Pan W . Imaging‐wide association study: integrating imaging endophenotypes in GWAS. NeuroImage. 2017;159:159‐169. doi:10.1016/j.neuroimage.2017.07.036 28736311 PMC5671364

[10] Knutson KA , Deng Y , Pan W . Implicating causal brain imaging endophenotypes in Alzheimer's disease using multivariable IWAS and GWAS summary data. NeuroImage. 2020;223:117347. doi:10.1016/j.neuroimage.2020.117347 32898681 PMC7778364

[11] Robert F , Pelletier J . Exploring the impact of single‐nucleotide polymorphisms on translation. Front Genet. 2018;9:507. doi:10.3389/fgene.2018.00507 30425729 PMC6218417

[12] Gamazon ER , Wheeler HE , Shah KP , et al. A gene‐based association method for mapping traits using reference transcriptome data. Nat Genet. 2015;47(9):1091‐1098. doi:10.1038/ng.3367 26258848 PMC4552594

[13] Mai J , Lu M , Gao Q , Zeng J , Xiao J . Transcriptome‐wide association studies: recent advances in methods, applications and available databases. Commun Biol. 2023;6(1):899. doi:10.1038/s42003-023-05279-y 37658226 PMC10474133

[14] Wainberg M , Sinnott‐Armstrong N , Mancuso N , et al. Opportunities and challenges for transcriptome‐wide association studies. Nat Genet. 2019;51(4):592‐599. doi:10.1038/s41588-019-0385-z 30926968 PMC6777347

[15] Barbeira AN , Dickinson SP , Bonazzola R , et al. Exploring the phenotypic consequences of tissue specific gene expression variation inferred from GWAS summary statistics. Nat Commun. 2018;9(1):1825. doi:10.1038/s41467-018-03621-1 29739930 PMC5940825

[16] Yuan SX , Li HT , Gu Y , Sun X . Brain‐specific gene expression and quantitative traits association analysis for mild cognitive impairment. Biomedicines. 2021;9(6):658. doi:10.3390/biomedicines9060658 34201204 PMC8229744

[17] Wang YH , Luo PP , Geng AY , et al. Identification of highly reliable risk genes for Alzheimer's disease through joint‐tissue integrative analysis. Front Aging Neurosci. 2023;15:1183119. doi:10.3389/fnagi.2023.1183119 37416324 PMC10320295

[18] Guo S , Yang J . Bayesian genome‐wide TWAS with reference transcriptomic data of brain and blood tissues identified 141 risk genes for Alzheimer's disease dementia. Alzheimers Res Ther. 2024;16:120. doi:10.1186/s13195-024-01488-7 38824563 PMC11144322

[19] Bycroft C , Freeman C , Petkova D , et al. The UK Biobank resource with deep phenotyping and genomic data. Nature. 2018;562(7726):203‐209. doi:10.1038/s41586-018-0579-z 30305743 PMC6786975

[20] Bledsoe X , Gamazon ER . A transcriptomic atlas of the human brain reveals genetically determined aspects of neuropsychiatric health. Am J Hum Genet. 2024;111(8):1559‐1572. doi:10.1016/j.ajhg.2024.06.002 38925120 PMC11339608

[21] Smith SM , Douaud G , Chen W , et al. An expanded set of genome‐wide association studies of brain imaging phenotypes in UK Biobank. Nat Neurosci. 2021;24(5):737‐745. doi:10.1038/s41593-021-00826-4 33875891 PMC7610742

[22] Miller KL , Alfaro‐Almagro F , Bangerter NK , et al. Multimodal population brain imaging in the UK Biobank prospective epidemiological study. Nat Neurosci. 2016;19(11):1523‐1536. doi:10.1038/nn.4393 27643430 PMC5086094

[23] Barbeira AN , Pividori M , Zheng J , Wheeler HE , Nicolae DL , Im HK . Integrating predicted transcriptome from multiple tissues improves association detection. PLOS Genet. 2019;15(1):e1007889. doi:10.1371/journal.pgen.1007889 30668570 PMC6358100

[24] Westman E , Aguilar C , Muehlboeck JS , Simmons A . Regional magnetic resonance imaging measures for multivariate analysis in Alzheimer's disease and mild cognitive impairment. Brain Topogr. 2013;26(1):9‐23. doi:10.1007/s10548-012-0246-x 22890700 PMC3536978

[25] Destrieux C , Fischl B , Dale A , Halgren E . Automatic parcellation of human cortical gyri and sulci using standard anatomical nomenclature. NeuroImage. 2010;53(1):1‐15. doi:10.1016/j.neuroimage.2010.06.010 20547229 PMC2937159

[26] Desikan RS , Segonne F , Fischl B , et al. An automated labeling system for subdividing the human cerebral cortex on MRI scans into gyral based regions of interest. Neuroimage. 2006;31(3):968‐980. 16530430 10.1016/j.neuroimage.2006.01.021

[27] Fischl B , Salat DH , Busa E , et al. Whole brain segmentation: automated labeling of neuroanatomical structures in the human brain. Neuron. 2002;33(3):341‐355. doi:10.1016/S0896-6273(02)00569-X 11832223

[28] Zhou D , Jiang Y , Zhong X , Cox NJ , Liu C , Gamazon ER . A unified framework for joint‐tissue transcriptome‐wide association and Mendelian randomization analysis. Nat Genet. 2020;52(11):1239‐1246. doi:10.1038/s41588-020-0706-2 33020666 PMC7606598

[29] Lee S , Lee DK . What is the proper way to apply the multiple comparison test? Korean J Anesthesiol. 2018;71(5):353‐360. doi:10.4097/kja.d.18.00242 30157585 PMC6193594

[30] Seto M , Dumitrescu L , Mahoney ER , et al. Multi‐omic characterization of brain changes in the vascular endothelial growth factor family during aging and Alzheimer's disease. Neurobiol Aging. 2023;126:25‐33. doi:10.1016/j.neurobiolaging.2023.01.010 36905877 PMC10106439

[31] Fujita M , Gao Z , Zeng L , et al. Cell subtype‐specific effects of genetic variation in the Alzheimer's disease brain. Nat Genet. 2024;56(4):605‐614. doi:10.1038/s41588-024-01685-y 38514782 PMC12288883

[32] He L , Davila‐Velderrain J , Sumida TS , Hafler DA , Kellis M , Kulminski AM . NEBULA is a fast negative binomial mixed model for differential or co‐expression analysis of large‐scale multi‐subject single‐cell data. Commun Biol. 2021;4(1):629. doi:10.1038/s42003-021-02146-6 34040149 PMC8155058

[33] Wu Y , Libby JB , Dumitrescu L , et al. Association of ten VEGF family genes with Alzheimer's disease endophenotypes at single cell resolution. Alzheimers Dement. 2024;21(2):e14419. doi:10.1002/alz.14419 39641382 PMC11848196

[34] Alfaro‐Almagro F , Jenkinson M , Bangerter NK , et al. Image processing and quality control for the first 10,000 brain imaging datasets from UK Biobank. Neuroimage. 2018;166:400‐424. doi:10.1016/j.neuroimage.2017.10.034 29079522 PMC5770339

[35] Zhang Y , Brady M , Smith S . Segmentation of brain MR images through a hidden Markov random field model and the expectation‐maximization algorithm. IEEE Trans Med Imaging. 2001;20(1):45‐57. doi:10.1109/42.906424 11293691

[36] Patenaude B , Smith SM , Kennedy DN , Jenkinson M . A Bayesian model of shape and appearance for subcortical brain segmentation. NeuroImage. 2011;56(3):907. doi:10.1016/j.neuroimage.2011.02.046 21352927 PMC3417233

[37] Thul PJ , Lindskog C . The human protein atlas: a spatial map of the human proteome. Protein Sci Publ Protein Soc. 2018;27(1):233‐244. doi:10.1002/pro.3307 PMC5734309 28940711

[38] Lu Q , Li B , Ou D , et al. A powerful approach to estimating annotation‐stratified genetic covariance via GWAS summary statistics. Am J Hum Genet. 2017;101(6):939‐964. doi:10.1016/j.ajhg.2017.11.001 29220677 PMC5812911

[39] Auton A , Abecasis GR , Altshuler DM , et al. A global reference for human genetic variation. Nature. 2015;526(7571):68‐74. doi:10.1038/nature15393 26432245 PMC4750478

[40] Zhang Y , Lu Q , Ye Y , et al. SUPERGNOVA: local genetic correlation analysis reveals heterogeneous etiologic sharing of complex traits. Genome Biol. 2021;22(1):262. doi:10.1186/s13059-021-02478-w 34493297 PMC8422619

[41] Berisa T , Pickrell JK . Approximately independent linkage disequilibrium blocks in human populations. Bioinformatics. 2016;32(2):283‐285. doi:10.1093/bioinformatics/btv546 26395773 PMC4731402

[42] Rigby RA , Stasinopoulos DM . Generalized Additive models for location, scale and shape. J R Stat Soc Ser C Appl Stat. 2005;54(3):507‐554. doi:10.1111/j.1467-9876.2005.00510.x

[43] Braak H , Alafuzoff I , Arzberger T , Kretzschmar H , Del Tredici K . Staging of Alzheimer disease‐associated neurofibrillary pathology using paraffin sections and immunocytochemistry. Acta Neuropathol (Berl). 2006;112(4):389‐404. 16906426 10.1007/s00401-006-0127-z PMC3906709

[44] Hollingworth P , Harold D , Sims R , et al. Common variants at ABCA7, MS4A6A /MS4A4E, EPHA1, CD33 and CD2AP are associated with Alzheimer's disease. Nat Genet. 2011;43(5):429‐435. 21460840 10.1038/ng.803 PMC3084173

[45] Naj AC , Jun G , Beecham GW , et al. Common variants at MS4A4/MS4A6E, CD2AP, CD33 and EPHA1 are associated with late‐onset Alzheimer's disease. Nat Genet. 2011;43(5):436‐441. doi:10.1038/ng.801 21460841 PMC3090745

[46] Gockley J , Montgomery KS , Poehlman WL , et al. Multi‐tissue neocortical transcriptome‐wide association study implicates 8 genes across 6 genomic loci in Alzheimer's disease. Genome Med. 2021;13:76. doi:10.1186/s13073-021-00890-2 33947463 PMC8094491

[47] Ferreira LK , Busatto GF . Neuroimaging in Alzheimer's disease: current role in clinical practice and potential future applications. Clinics. 2011;66(Suppl 1):19‐24. doi:10.1590/S1807-59322011001300003 21779719 PMC3118433

[48] Kim J , Jeong M , Stiles WR , Choi HS . Neuroimaging modalities in Alzheimer's disease: diagnosis and clinical features. Int J Mol Sci. 2022;23(11):6079. doi:10.3390/ijms23116079 35682758 PMC9181385

[49] Lebedeva AK , Westman E , Borza T , et al. MRI‐based classification models in prediction of mild cognitive impairment and dementia in late‐life depression. Front Aging Neurosci. 2017;9:13. doi:10.3389/fnagi.2017.00013 28210220 PMC5288688

[50] Pecher H , Storch M , Beyer F , et al. Hypothalamic atrophy and structural covariance in amnestic mild cognitive impairment and Alzheimer's dementia. NeuroImage Clin. 2024;44:103687. doi:10.1016/j.nicl.2024.103687 39406040 PMC11525751

[51] Ghanbari M , Ikram MA , de Looper HWJ , et al. Genome‐wide identification of microRNA‐related variants associated with risk of Alzheimer's disease. Sci Rep. 2016;6(1):28387. doi:10.1038/srep28387 27328823 PMC4916596

[52] Scahill RI , Schott JM , Stevens JM , Rossor MN , Fox NC . Mapping the evolution of regional atrophy in Alzheimer's disease: unbiased analysis of fluid‐registered serial MRI. Proc Natl Acad Sci U S A. 2002;99(7):4703‐4707. doi:10.1073/pnas.052587399 11930016 PMC123711

[53] Yue W . The structural changes of frontal subregions and their correlations with cognitive impairment in patients with Alzheimer's disease. J Integr Neurosci. 2023;22(4):99. doi:10.31083/j.jin2204099 37519164

[54] Lorenz A , Sathe A , Zaras D , et al. The effect of Alzheimer's disease genetic factors on limbic white matter microstructure. Alzheimers Dement. 2025;21(4):e70130. doi:10.1002/alz.70130 40219815 PMC11992597

[55] Peter C , Sathe A , Shashikumar N , et al. White matter abnormalities and cognition in aging and alzheimer disease. JAMA Neurol. 2025;82(8):825‐836. Published online 2025. doi:10.1001/jamaneurol.2025.1601 40513084 PMC12150229

[56] Zammit AR , Ezzati A , Zimmerman ME , Lipton RB , Lipton ML , Katz MJ . Roles of hippocampal subfields in verbal and visual episodic memory. Behav Brain Res. 2017;317:157‐162. doi:10.1016/j.bbr.2016.09.038 27646772 PMC6343125

[57] Rao YL , Ganaraja B , Murlimanju BV , Joy T , Krishnamurthy A , Agrawal A . Hippocampus and its involvement in Alzheimer's disease: a review. 3 Biotech. 2022;12(2):55. doi:10.1007/s13205-022-03123-4 PMC8807768 35116217

[58] Apostolova LG , Mosconi L , Thompson PM , et al. Subregional hippocampal atrophy predicts Alzheimer's dementia in the cognitively normal. Neurobiol Aging. 2010;31(7):1077‐1088. doi:10.1016/j.neurobiolaging.2008.08.008 18814937 PMC2873083

[59] Mueller SG , Stables L , Du AT , et al. Measurement of hippocampal subfields and age‐related changes with high resolution MRI at 4 T. Neurobiol Aging. 2007;28(5):719‐726. doi:10.1016/j.neurobiolaging.2006.03.007 16713659 PMC1820772

[60] West MJ , Kawas CH , Stewart WF , Rudow GL , Troncoso JC . Hippocampal neurons in pre‐clinical Alzheimer's disease. Neurobiol Aging. 2004;25(9):1205‐1212. doi:10.1016/j.neurobiolaging.2003.12.005 15312966

[61] Krantic S , Isorce N , Mechawar N , et al. Hippocampal GABAergic neurons are susceptible to amyloid‐β toxicity in vitro and are decreased in number in the Alzheimer's disease TgCRND8 mouse model. J Alzheimers Dis JAD. 2012;29(2):293‐308. doi:10.3233/JAD-2011-110830 22232004

[62] Haegert DG . Analysis of the threshold liability model provides new understanding of causation in autoimmune diseases. Med Hypotheses. 2004;63(2):257‐261. doi:10.1016/j.mehy.2004.02.015 15236786

[63] Jansen IE , van der Lee SJ , Gomez‐Fonseca D , et al. Genome‐wide meta‐analysis for Alzheimer's disease cerebrospinal fluid biomarkers. Acta Neuropathol (Berl). 2022;144(5):821‐842. doi:10.1007/s00401-022-02454-z 36066633 PMC9547780

[64] Wu Y , Sun Z , Zheng Q , et al. Pervasive biases in proxy genome‐wide association studies based on parental history of Alzheimer's disease. Nat Genet. 2024;56(12):2696‐2703. doi:10.1038/s41588-024-01963-9 39496879 PMC11929606

