# Supplementary material for: Neuroimaging PheWAS and molecular phenotyping implicate PSMC3 in Alzheimer's disease
Source: Alzheimers Dement. 2026 Feb 24;22(2):e71217. doi: 10.1002/alz.71217 (PMC12930104; doi:10.1002/alz.71217)
Supplement: Supplementary file 2 — Supporting Information [file ALZ-22-e71217-s002.docx]

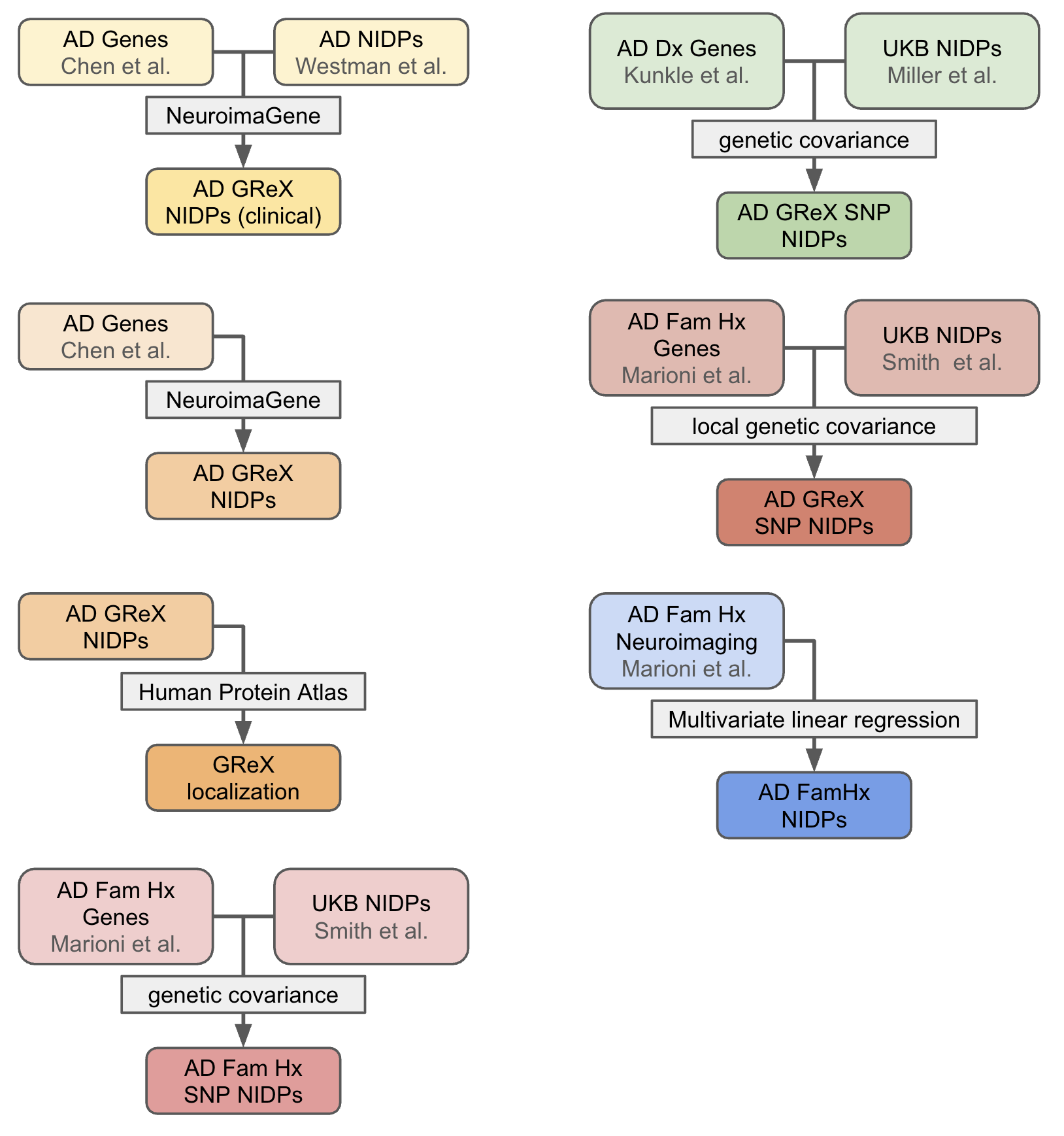


Supplemental Figure 1: Overview of all analyses performed in this paper with resources and results in color and primary analytical method indicated in greyscale. AD – Alzheimer’s Disease; NIDP – Neuroimaging derived Phenotype; GReX – genetically regulated gene expression; Fam Hx – family history; SNP – single nucleotide polymorphism; Dx – diagnosis


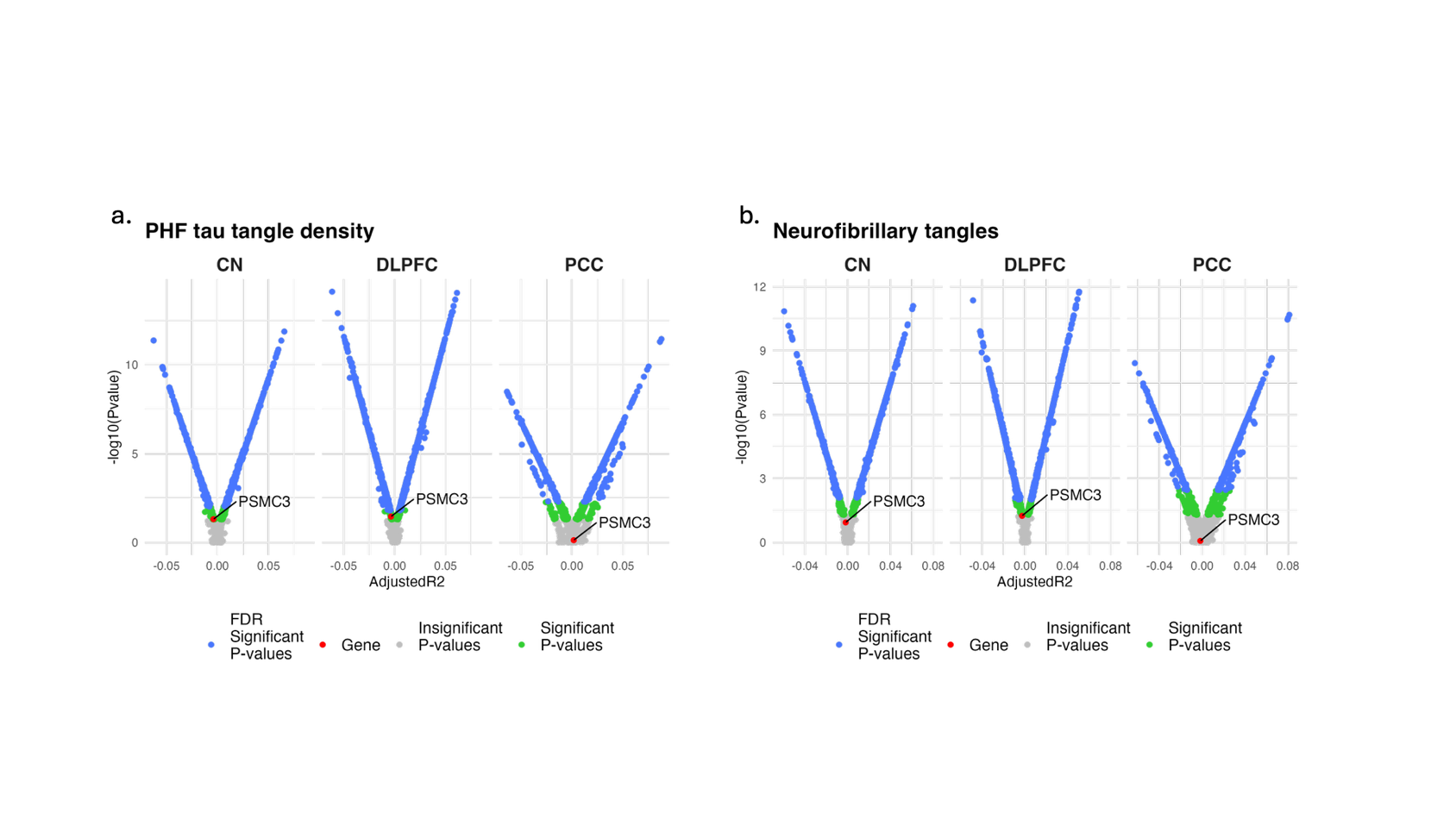


Figure S2: *PSMC3* expression levels from bulk RNA sequencing in ROSMAP associated with AD tau pathology assessed by (a) immunohistochemistry staining and (b) silver staining. The x-axis in the volcano plot represents the adjusted R^2^ value derived from the regression analyses. The y-axis represents the -log_10_ *P-*values for significance. The blue dots represent FDR significant *P-*values (*P*_FDR_ < 0.05), the green dots represent nominally significant *P-*values, and the grey dots represent insignificant *P-*values. The red dot indicates *PSMC3.* CN = caudate nucleus. DLPFC = dorsolateral prefrontal cortex. PCC = posterior cingulate cortex.


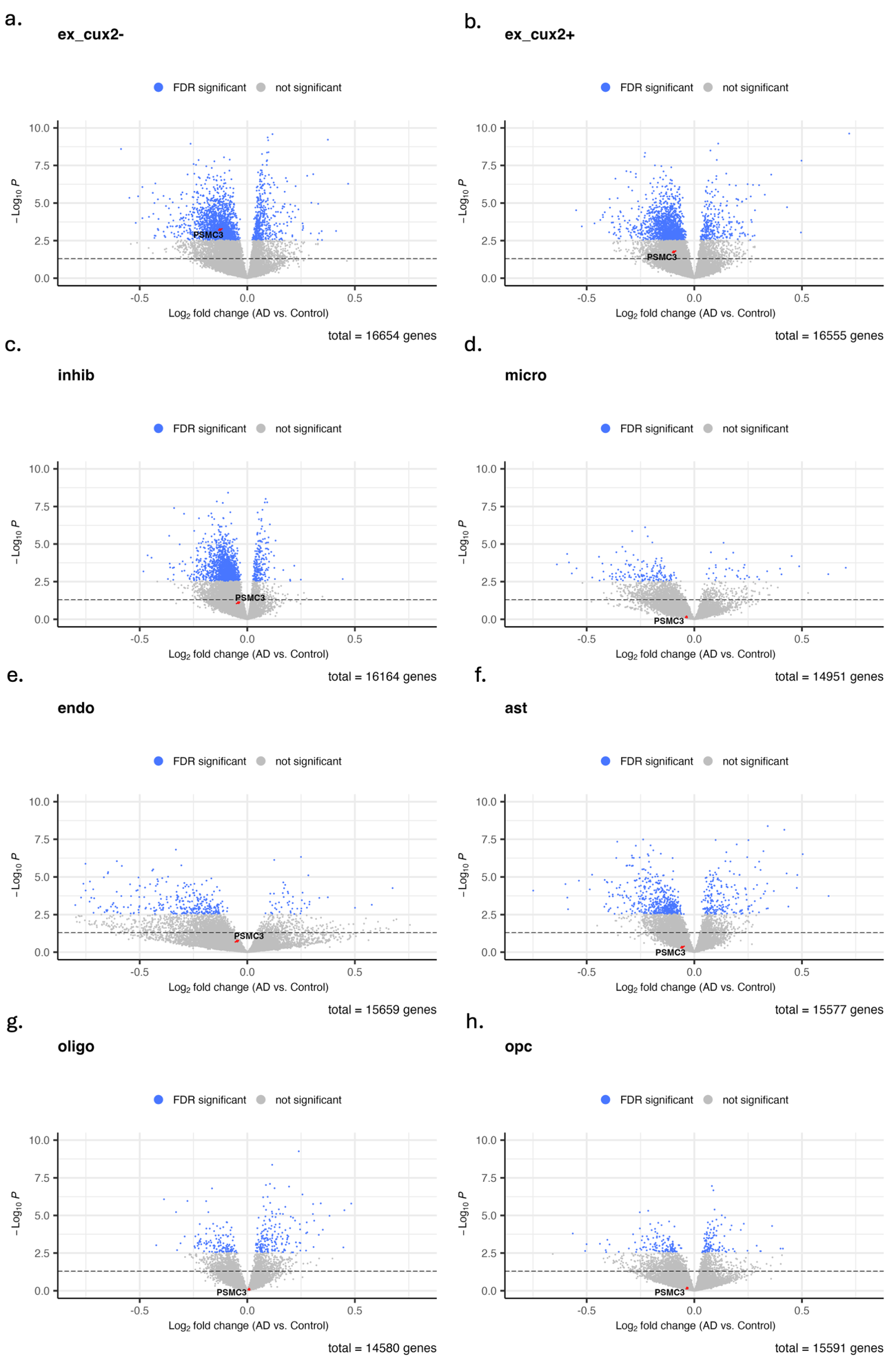


Figure S3: *PSMC3* expression levels from single-nucleus RNA sequencing in ROSMAP associated with AD case/control status across eight DLPFC cell types. The x-axis represents the log-fold change of *PSMC3* expression between AD cases and normal controls. The y-axis represents the -log_10_ *P-*values for significance. The blue dots represent FDR significant *P-*values (*P*_FDR_ < 0.05), and the grey dots represent insignificant *P-*values. The red arrow indicated *PSMC3.* ex = excitatory neurons, inhib = inhibitory neurons, micro = microglia, endo = endothelial cells, ast = astrocytes*,* oligo = oligodendrocytes, opc = oligodendrocyte progenitor cells*.*


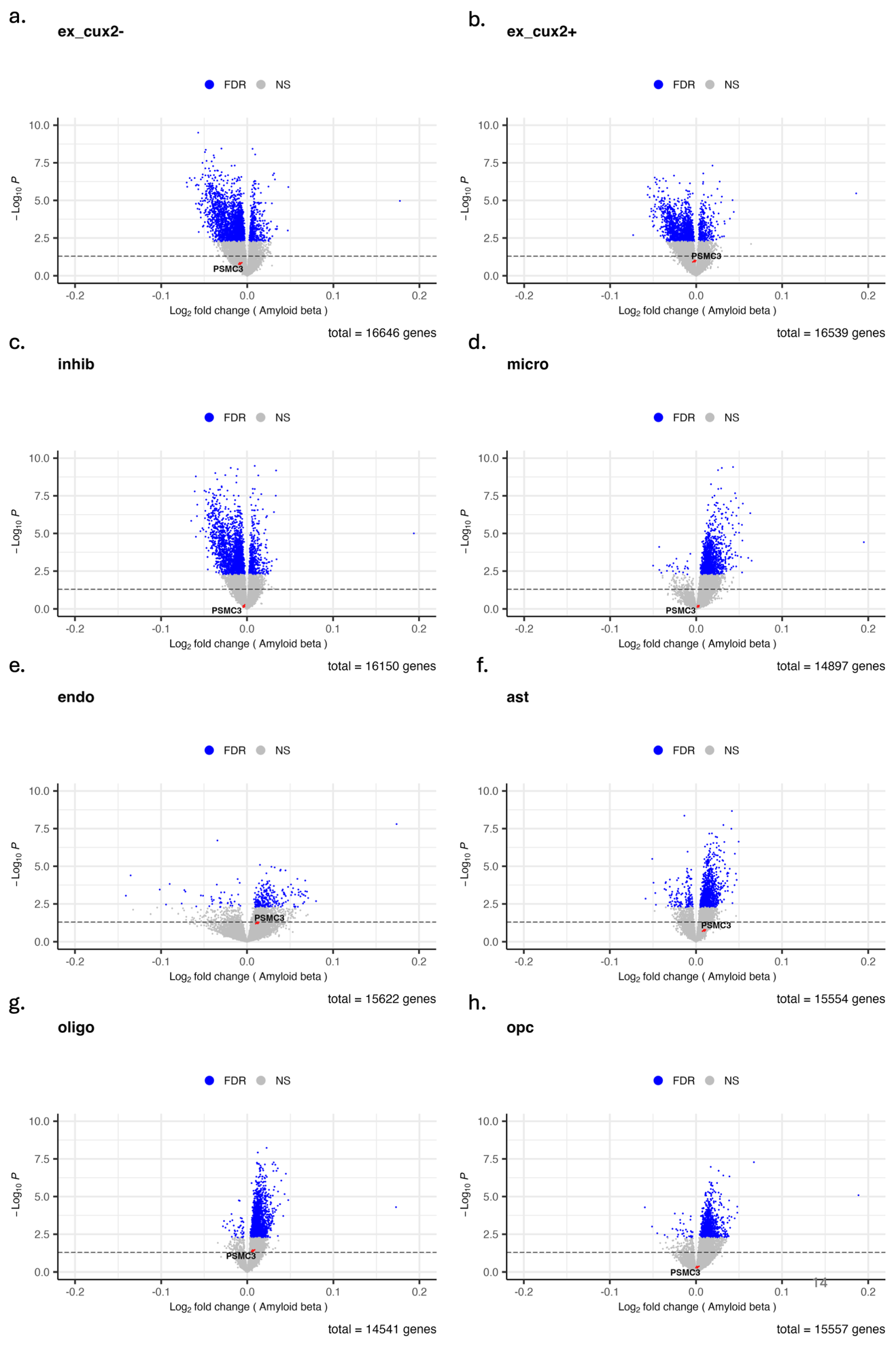


Figure S4: *PSMC3* expression levels from single-nucleus RNA sequencing in ROSMAP associated with amyloid beta across eight DLPFC cell types. The x-axis represents the log-fold change of *PSMC3* expression in relation to amyloid beta deposition. The y-axis represents the -log_10_ *P-*values for significance. The blue dots represent FDR significant *P-*values (*P*_FDR_ < 0.05), and the grey dots represent insignificant *P-*values. The red arrow indicated *PSMC3.* ex = excitatory neurons, inhib = inhibitory neurons, micro = microglia, endo = endothelial cells, ast = astrocytes*,* oligo = oligodendrocytes, opc = oligodendrocyte progenitor cells.


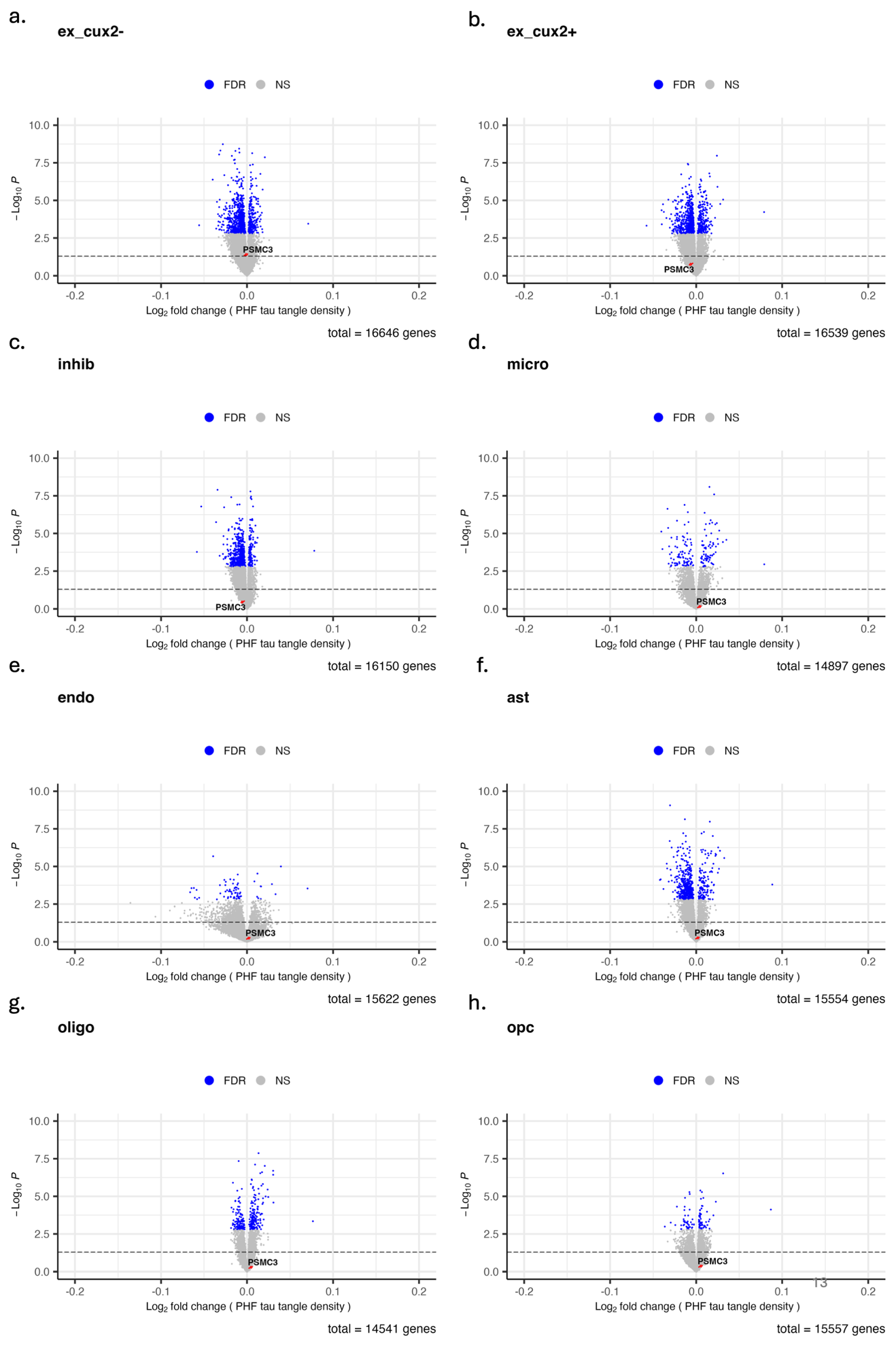


Figure S5: *PSMC3* expression levels from single-nucleus RNA sequencing in ROSMAP associated with tau tangles across eight DLPFC cell types. The x-axis represents the log-fold change of *PSMC3* expression in relation to tau tangles deposition. The y-axis represents the -log_10_ *P-*values for significance. The blue dots represent FDR significant *P-*values (*P*_FDR_ < 0.05), and the grey dots represent insignificant *P-*values. The red arrow indicated *PSMC3.* ex = excitatory neurons, inhib = inhibitory neurons, micro = microglia, endo = endothelial cells, ast = astrocytes*,* oligo = oligodendrocytes, opc = oligodendrocyte progenitor cells.


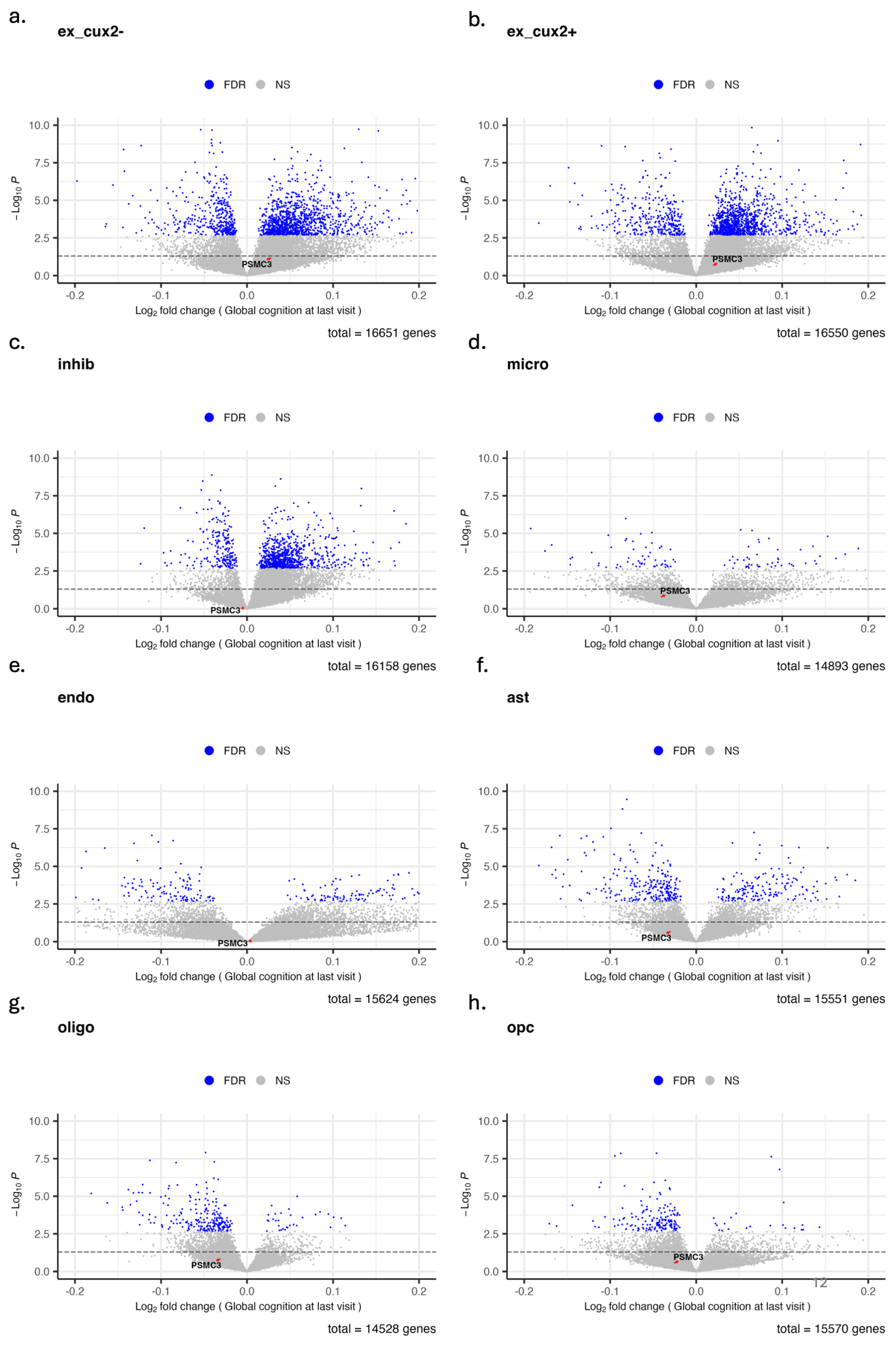


Figure S6: *PSMC3* expression levels from single-nucleus RNA sequencing in ROSMAP associated with global cognition score at last visit across eight DLPFC cell types. The x-axis represents the log-fold change of *PSMC3* expression in relation to the global cognition score at the last visit. The y-axis represents the -log_10_ *P-*values for significance. The blue dots represent FDR significant *P-*values (*P*_FDR_ < 0.05), and the grey dots represent insignificant *P-*values. The red arrow indicated *PSMC3.* ex = excitatory neurons, inhib = inhibitory neurons, micro = microglia, endo = endothelial cells, ast = astrocytes*,* oligo = oligodendrocytes, opc = oligodendrocyte progenitor cells.


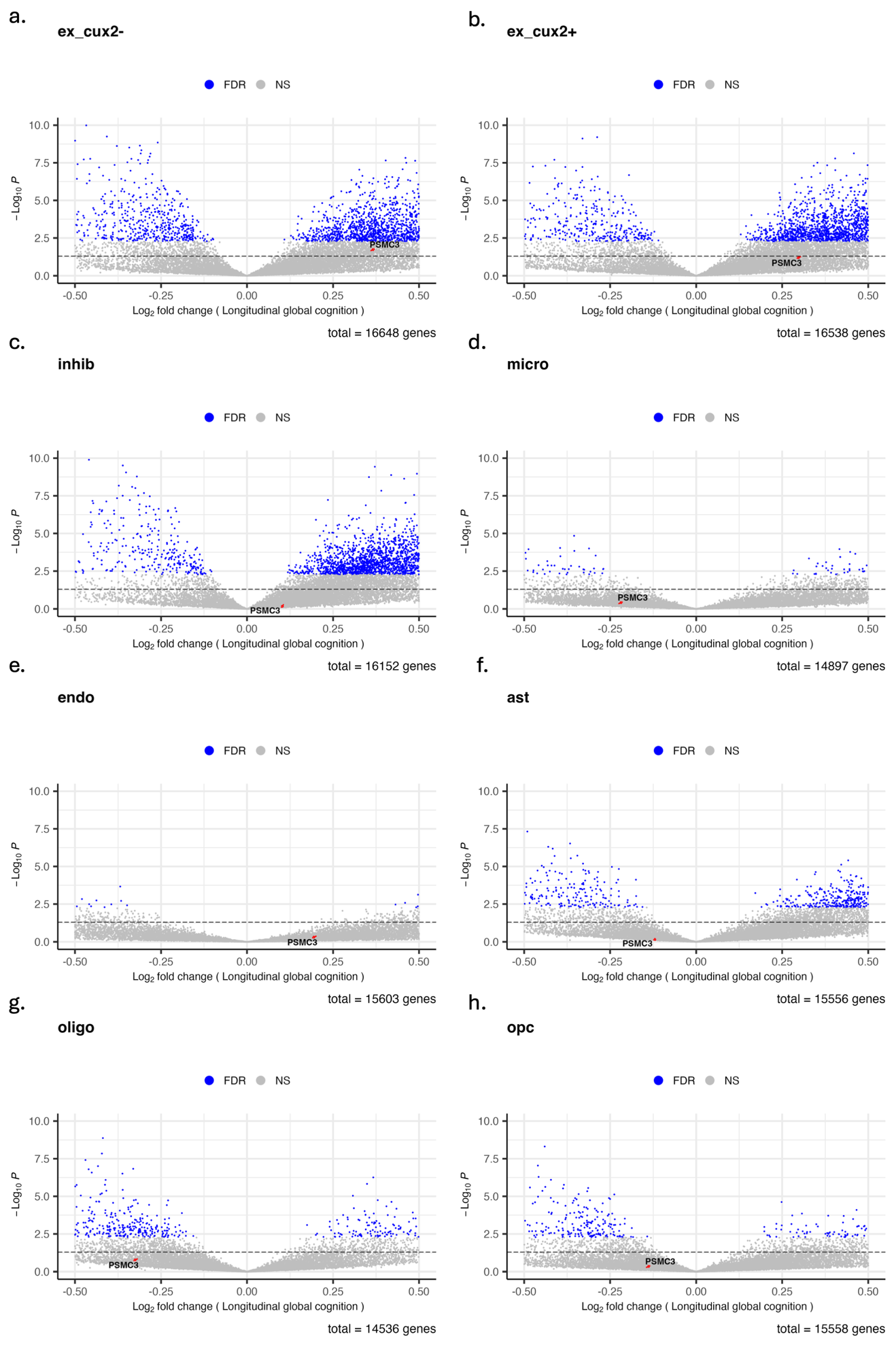


Figure S7: *PSMC3* expression levels from single-nucleus RNA sequencing in ROSMAP associated with longitudinal global cognition trajectory across eight DLPFC cell types. The x-axis represents the log-fold change of *PSMC3* expression in relation to the longitudinal global cognition trajectory. The y-axis represents the -log_10_ *P-*values for significance. The blue dots represent FDR significant *P-*values (*P*_FDR_ < 0.05), and the grey dots represent insignificant *P-*values. The red arrow indicated *PSMC3.* ex = excitatory neurons, inhib = inhibitory neurons, micro = microglia, endo = endothelial cells, ast = astrocytes*,* oligo = oligodendrocytes, opc = oligodendrocyte progenitor cells.


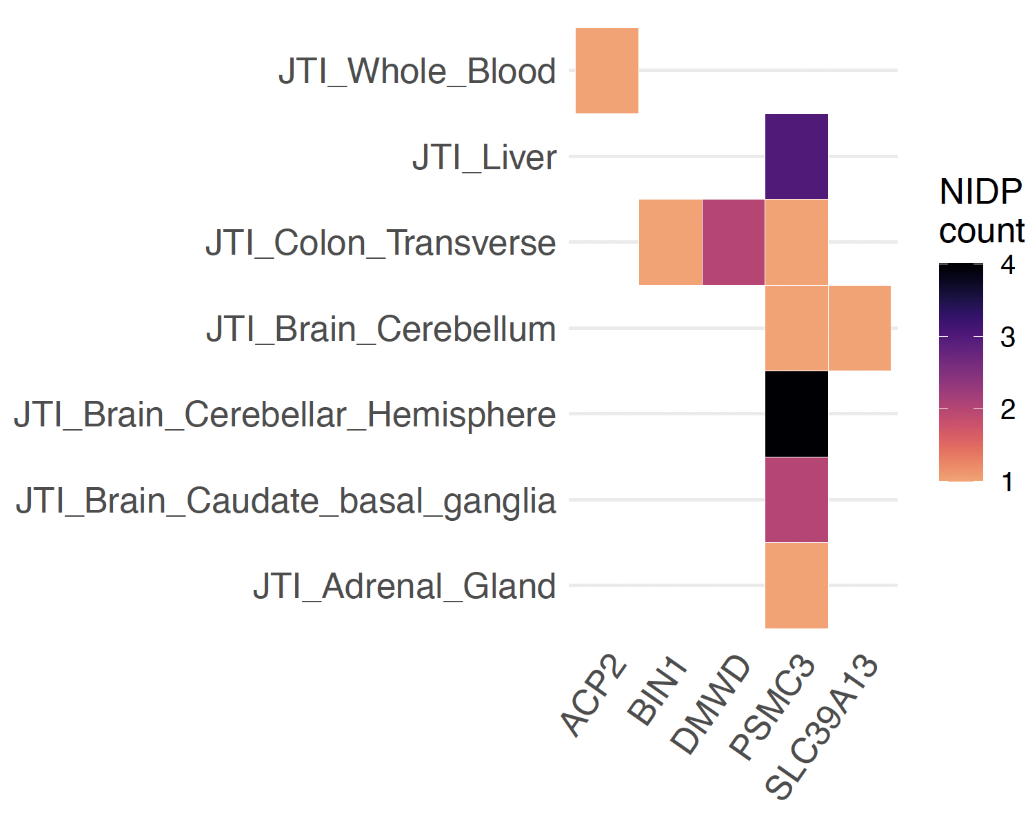


Figure S8: Tile plot detailing statistically significant associations between AD GReX measures (x-axis) and subcortical and cortical NIDPs derived from the FIRST and FAST neuroimaging MRI segmentation protocols. Associations are distributed across the tissue models in which the association was found to be significant (y-axis). These data are for the image-agnostic neuroimaging TWAS analysis. The color of each tile represents the number of NIDPs with which the gene is associated in each particular tissue model. Statistical significance was assessed via TWAS with FDR thresholding and heterogeneity adjustment applied via the MR-JTI causal inference framework. NIDP – neuroimaging derived phenotype, JTI – joint tissue imputation; MRI – magnetic resonance imaging


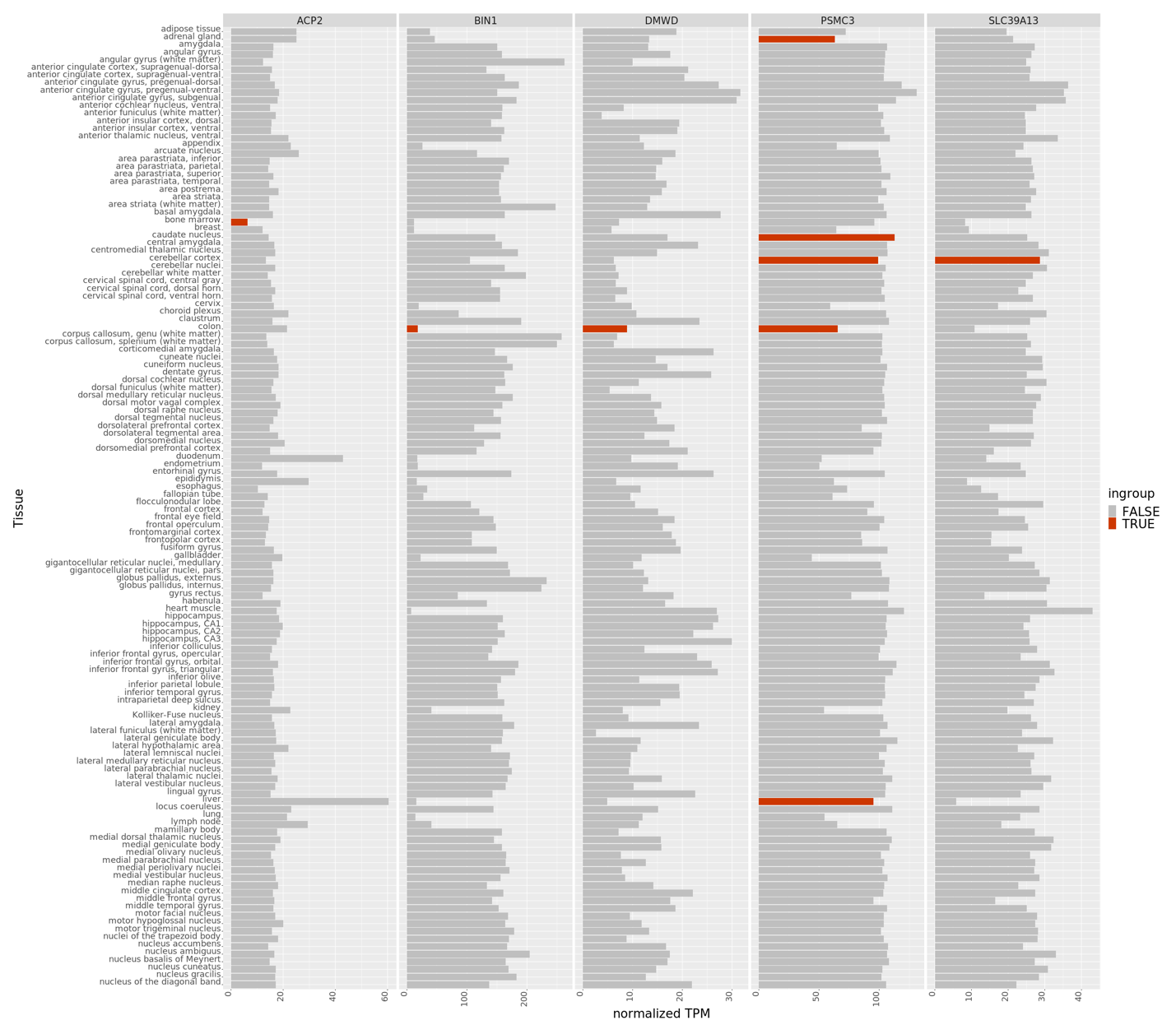


Figure S9: Expression values in normalized transcripts per million of genes associated with neuroimaging features through expression. Data reflect gene expression across all tissues in the Human Protein Atlas. Orange bars represent tissues in which the gene is significantly associated with an AD-derived neuroimaging measure. TPM – transcripts per million


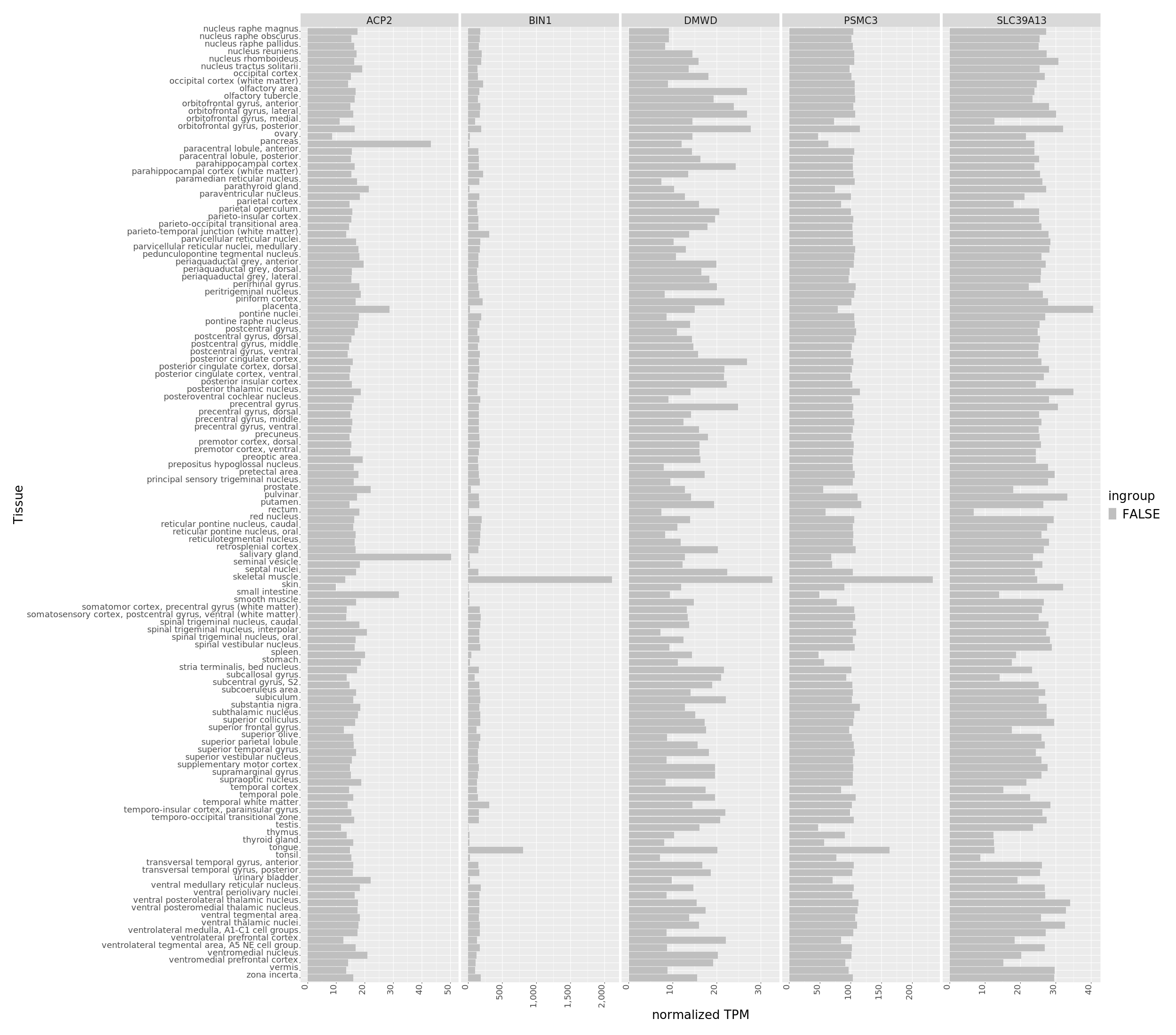


Figure S10: Expression values in normalized transcripts per million of genes associated with neuroimaging features derived from clinical AD studies through expression. Data reflect gene expression across all tissues in the Human Protein Atlas. Orange bars represent tissues in which the gene is significantly associated with an AD-derived neuroimaging measure. TPM – transcripts per million


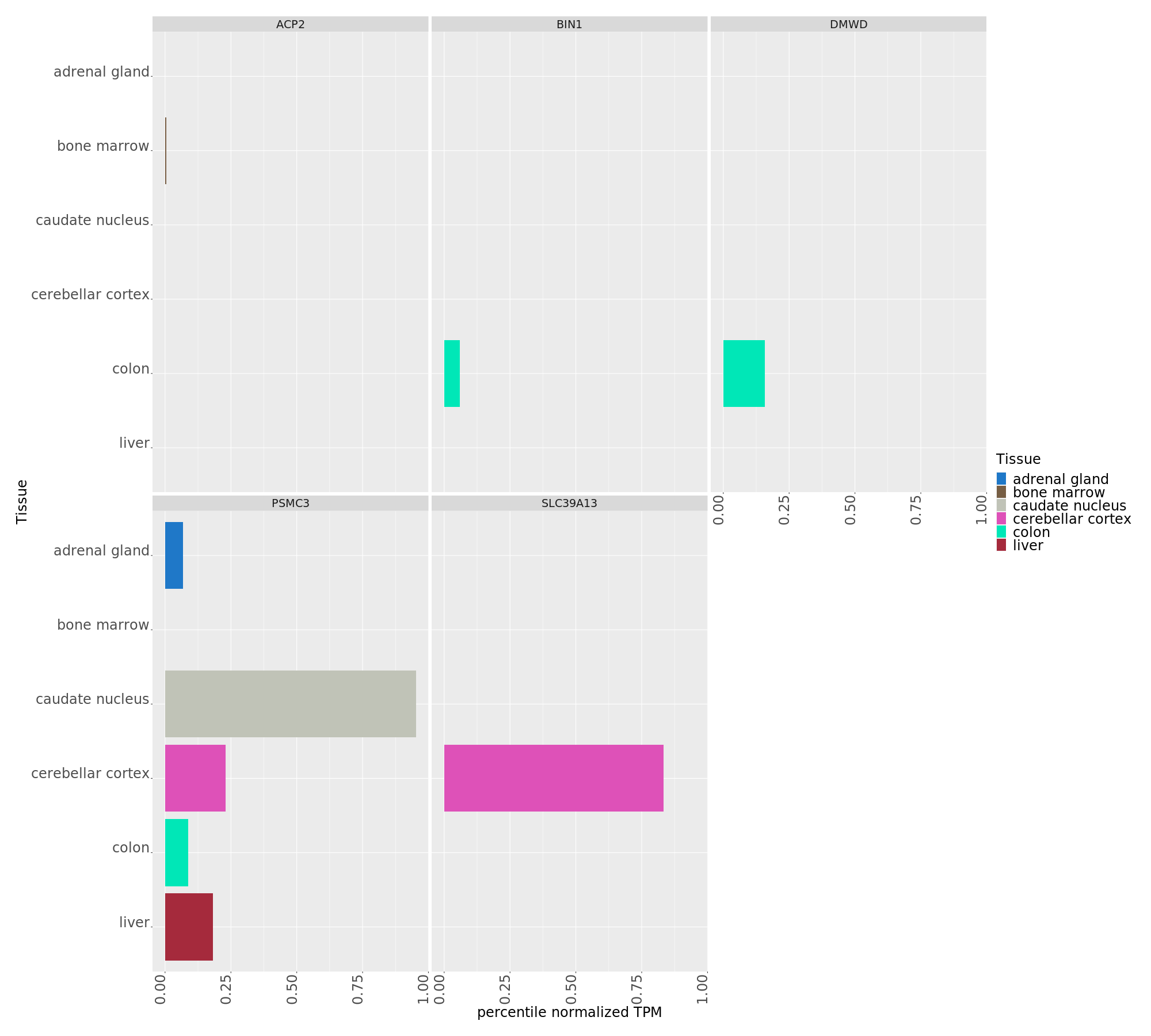


Figure S11: Tissue specific expression data of transcripts derived from the image-agnostic analysis of Alzheimer's neuroimaging data. Expression data were derived from RNA-sequencing measurements with values expressed by normalized transcripts per million (TPM) and ranked by percentile.


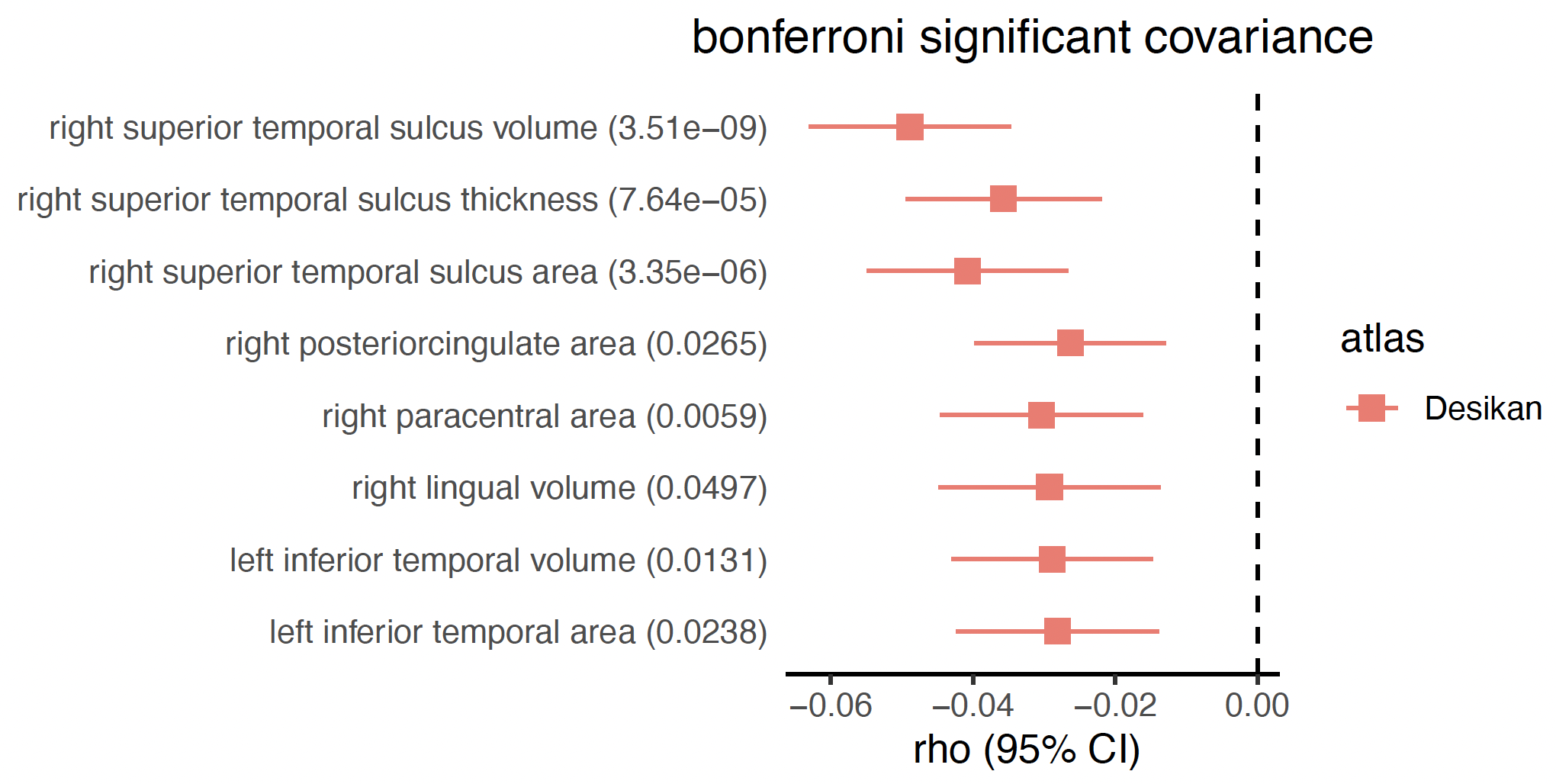


Figure S12: Bonferroni significant global genetic covariance between Neuroimaging Derived Phenotypes and family history of dementia across all Desikan T1-derived NIDPs. Genetic covariance was calculated using the GeNetic cOVariance Analyzer (GNOVA).


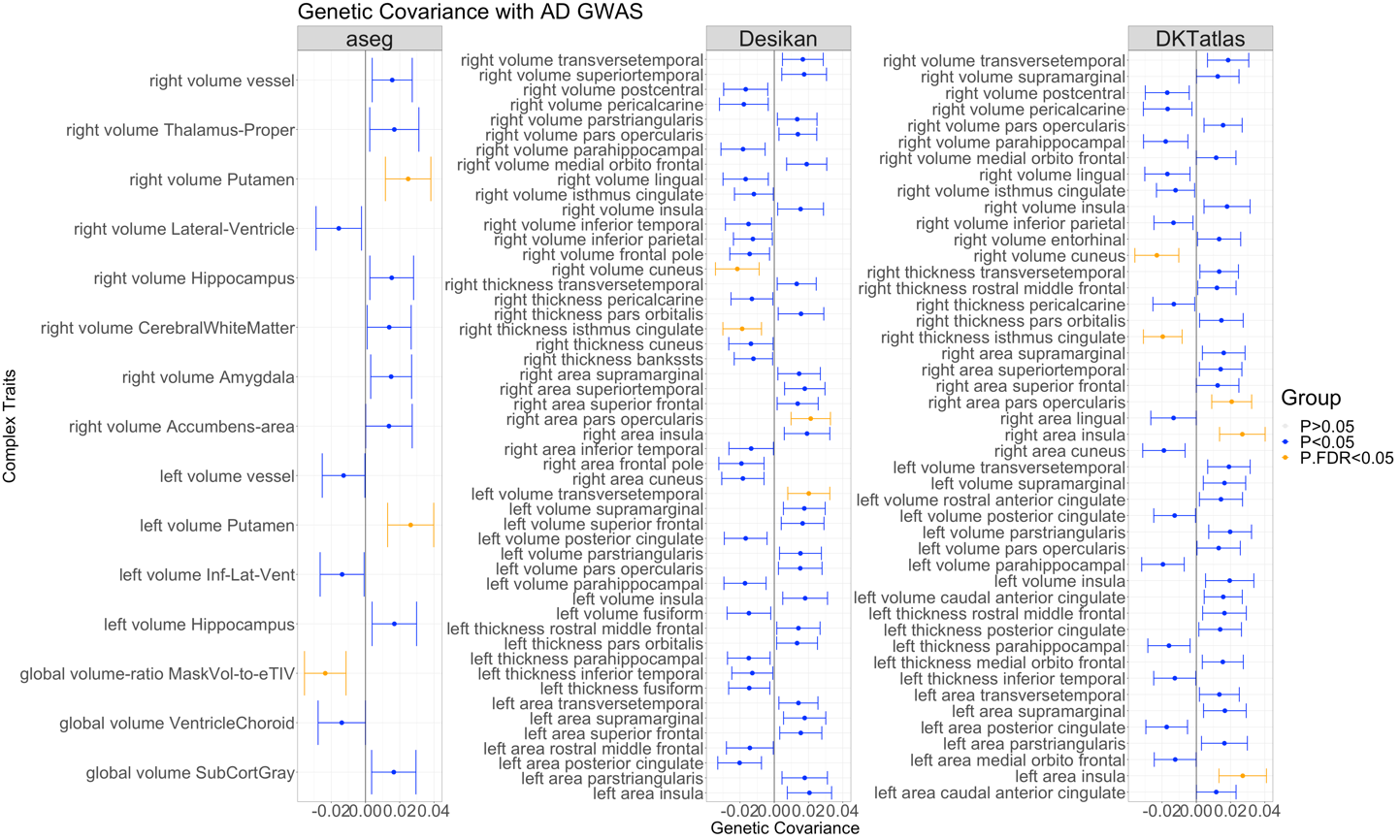


Figure S13: Genetic covariance results performed using GeNetic cOVariance Analyzer (GNOVA) between Neuroimaging Derived Phenotypes (NIDPs), processed using aseg, Desikan, and DKT atlases, and clinical Alzheimer's Disease status indicated by Kunkle et al.’s study. The x-axis represents the genetic covariance estimates. The y-axis demonstrates the NIDPs. Each facet represents the results from a different atlas. The FDR-significant results are colored orange, and the nominally significant results are colored blue. The non-significant results are not shown here. FDR – false discovery rate; NIDP – neuroimaging-derived phenotypes


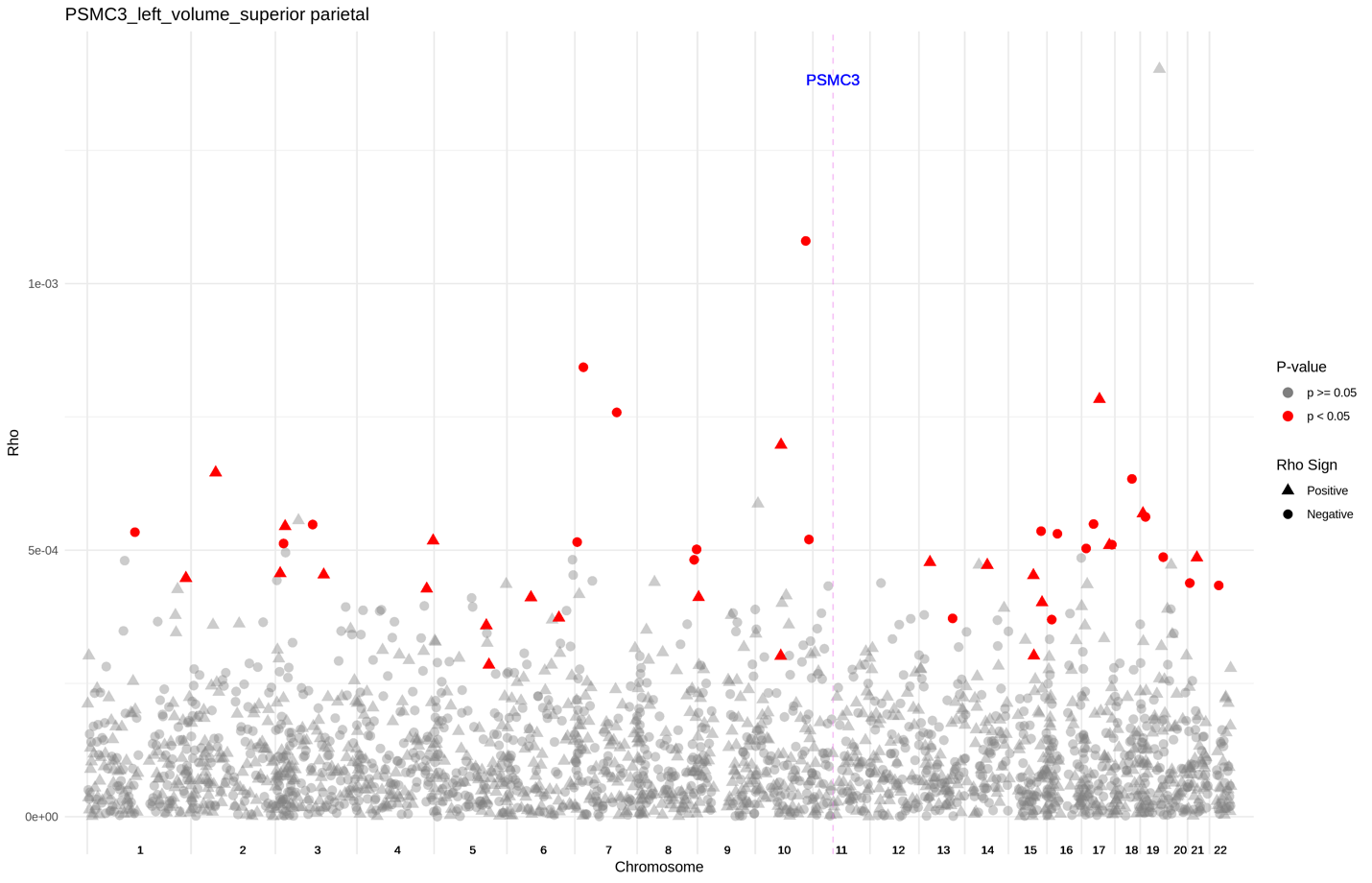


Figure S14: Genome-wide results from the local genetic covariance analysis between the family history of dementia GWAS by Marioni et al. and the listed NIDP from the image-directed analysis result prior to the Mendelian randomization (MR) test. Each point represents a single locus, with the y-axis location indicating the covariance between SNPs effect sizes in that region. The color of the point represents significance, with red indicating nominal significance. As each NIDP was identified according to associations with AD-genes, we overlaid the location of the associated gene onto the superGNOVA findings. Significant loci occurring within 2 megabases of the gene of interest are emphasized with a larger size.


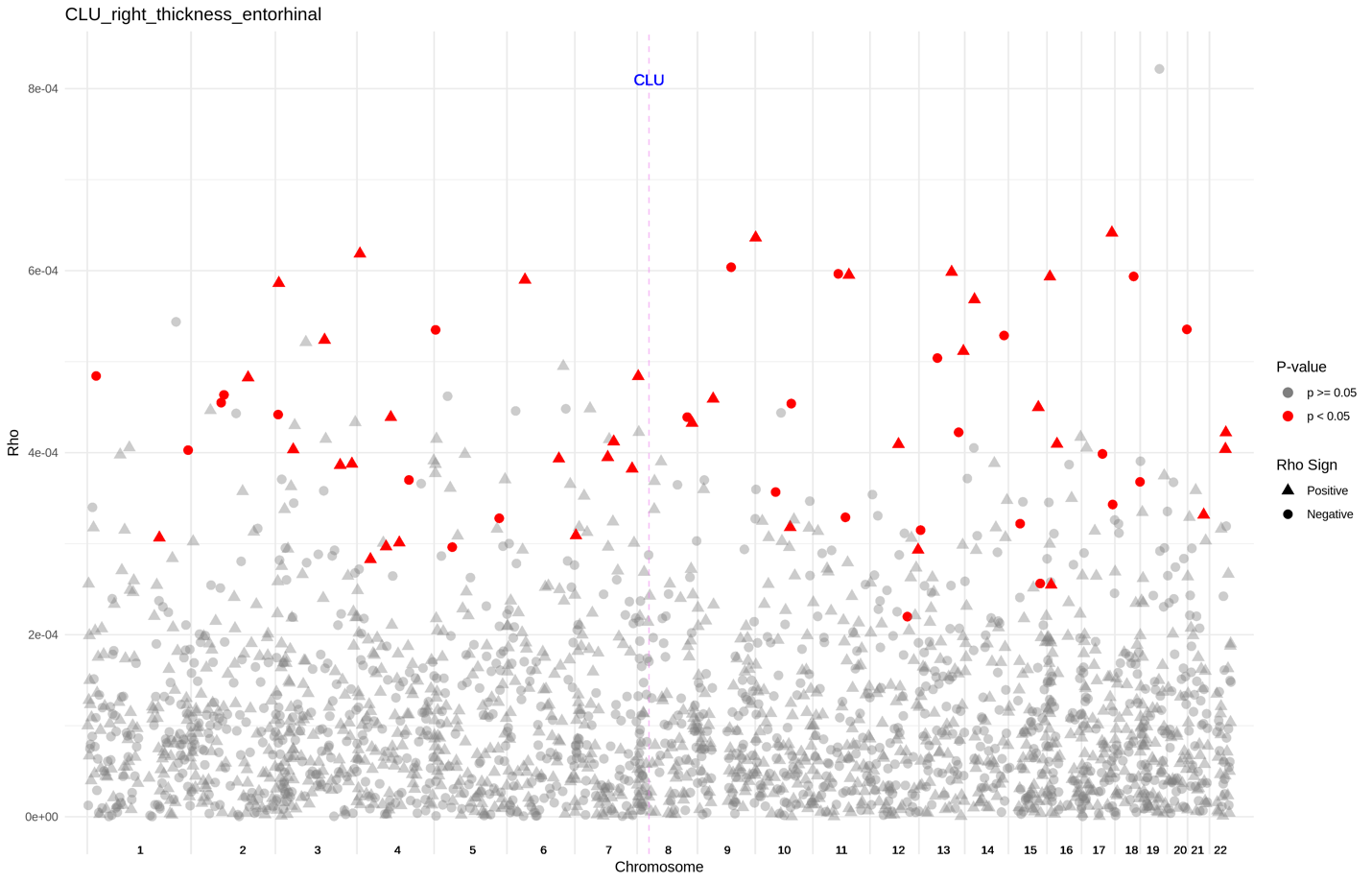


Figure S15: Genome-wide results from the local genetic covariance analysis between the family history of dementia GWAS by Marioni et al. and the listed NIDP from the image-directed analysis result prior to the MR test. Each point represents a single locus, with the y-axis location indicating the covariance between SNPs effect sizes in that region. The color of the point represents significance, with red indicating nominal significance. As each NIDP was identified according to associations with AD-genes, we overlaid the location of the associated gene onto the superGNOVA findings. Significant loci occurring within 2 megabases of the gene of interest are emphasized with a larger size.


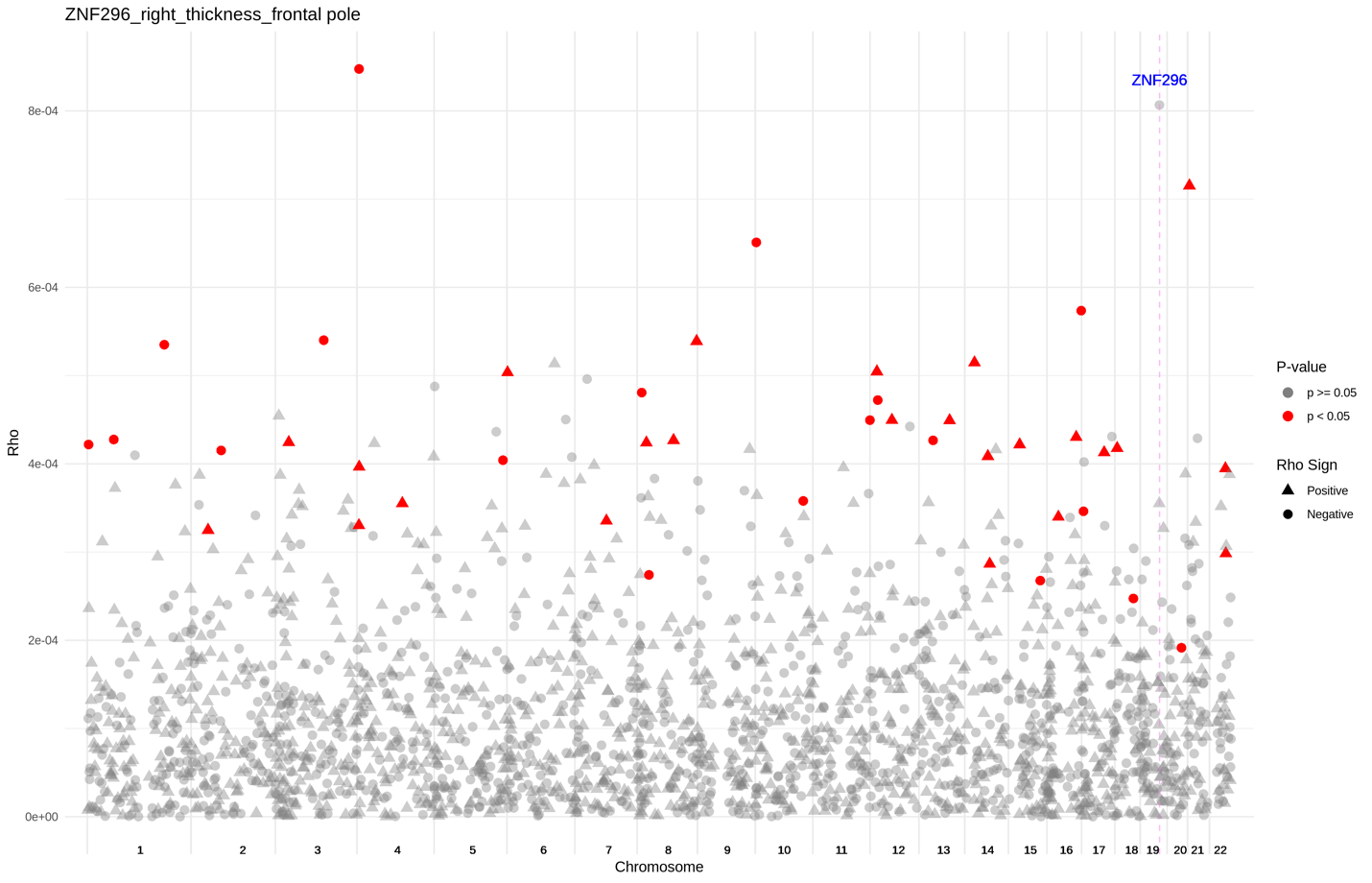


Figure S16: Genome-wide results from the local genetic covariance analysis between the family history of dementia GWAS by Marioni et al. and the listed NIDP from the image-directed analysis result prior to the MR test. Each point represents a single locus, with the y-axis location indicating the covariance between SNPs effect sizes in that region. The color of the point represents significance, with red indicating nominal significance. As each NIDP was identified according to associations with AD-genes, we overlaid the location of the associated gene onto the superGNOVA findings. Significant loci occurring within 2 megabases of the gene of interest are emphasized with a larger size.


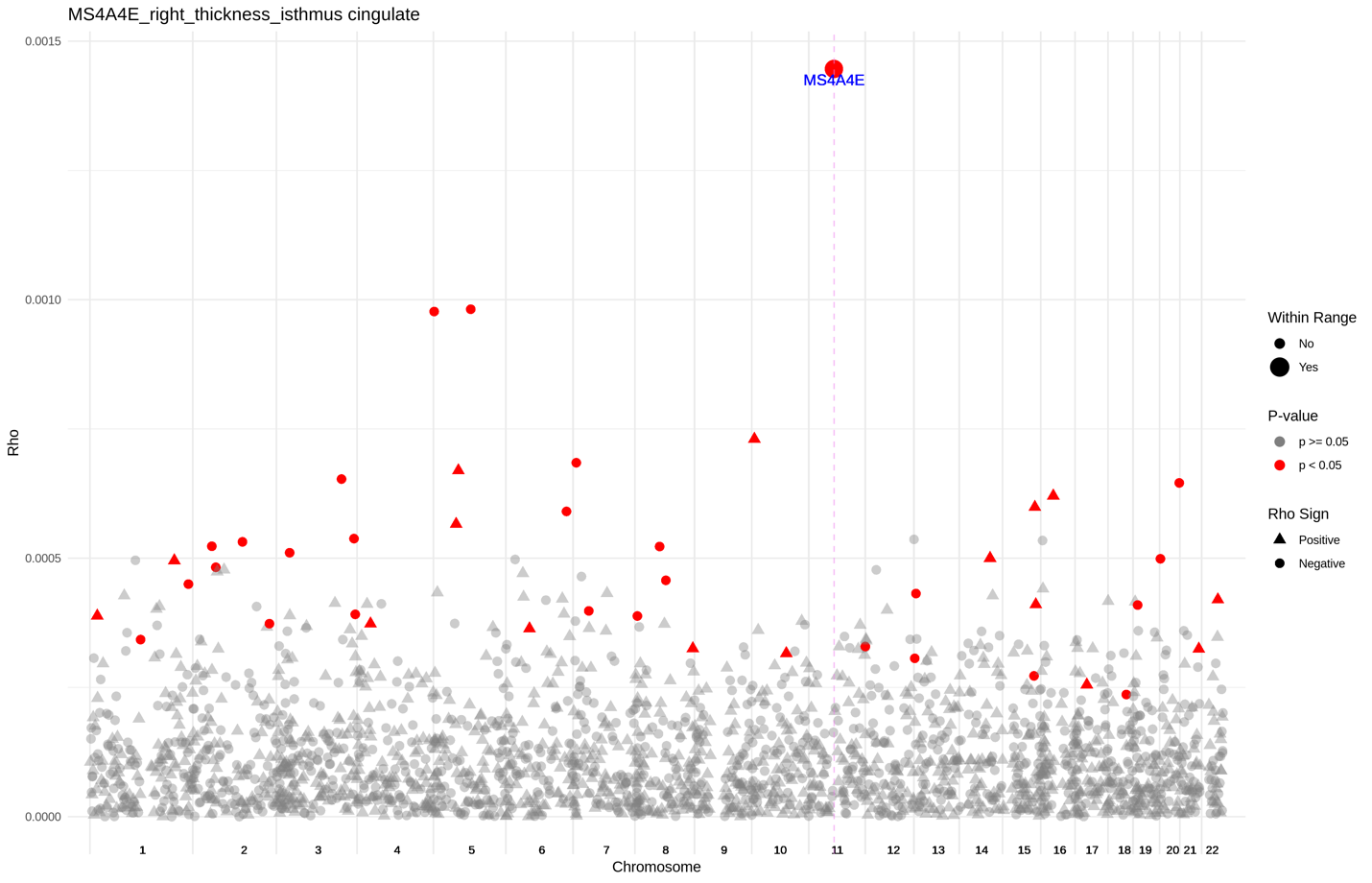


Figure S17: Genome-wide results from the local genetic covariance analysis between the family history of dementia GWAS by Marioni et al. and the listed NIDP from the image-directed analysis result prior to the MR test. Each point represents a single locus, with the y-axis location indicating the covariance between SNPs effect sizes in that region. The color of the point represents significance, with red indicating nominal significance. As each NIDP was identified according to associations with AD-genes, we overlaid the location of the associated gene onto the superGNOVA findings. Significant loci occurring within 2 megabases of the gene of interest are emphasized with a larger size.


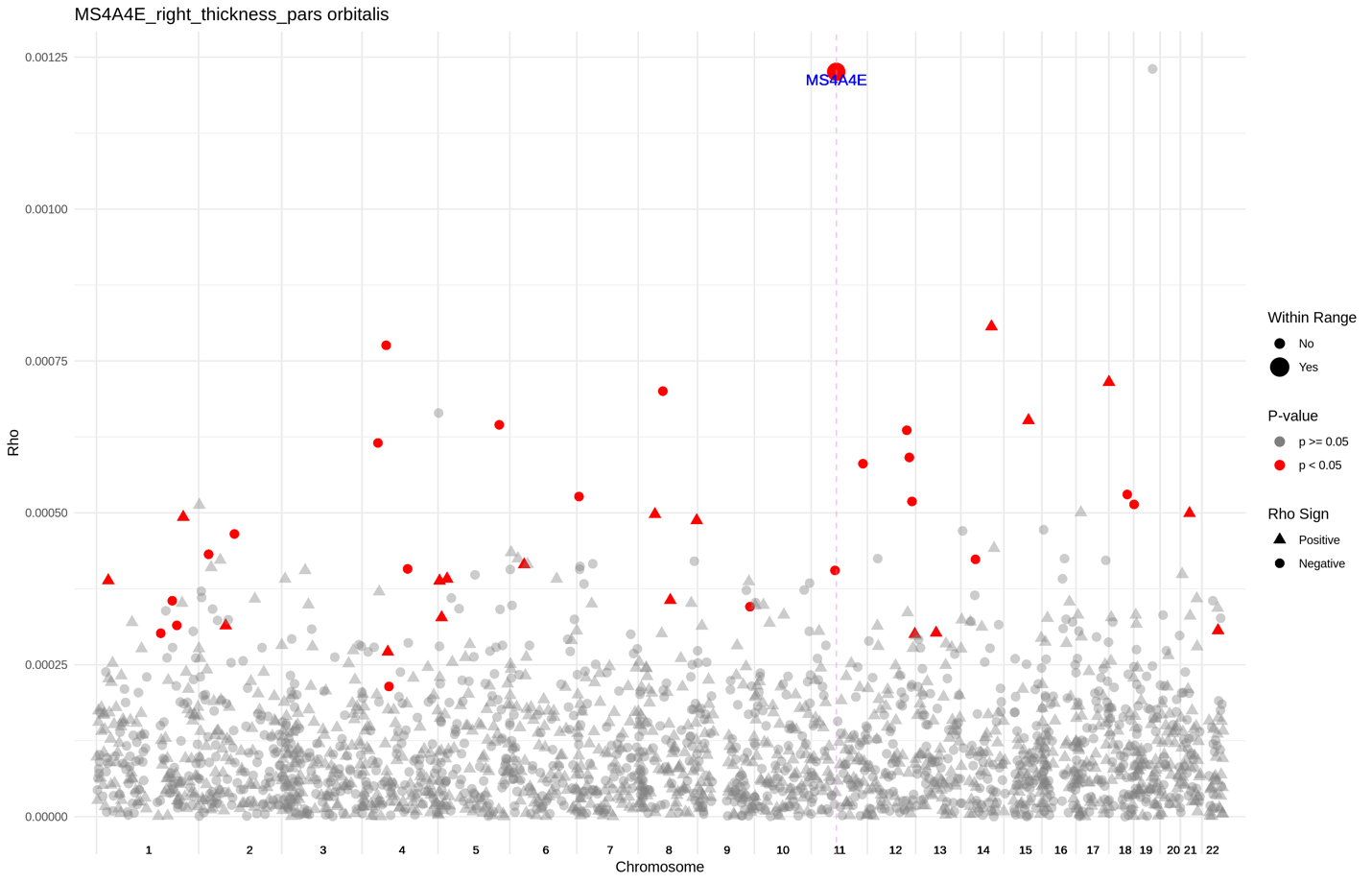


Figure S18: Genome-wide results from the local genetic covariance analysis between the family history of dementia GWAS by Marioni et al. and the listed NIDP from the image-directed analysis result prior to the MR test. Each point represents a single locus, with the y-axis location indicating the covariance between SNPs effect sizes in that region. The color of the point represents significance, with red indicating nominal significance. As each NIDP was identified according to associations with AD-genes, we overlaid the location of the associated gene onto the superGNOVA findings. Significant loci occurring within 2 megabases of the gene of interest are emphasized with a larger size.


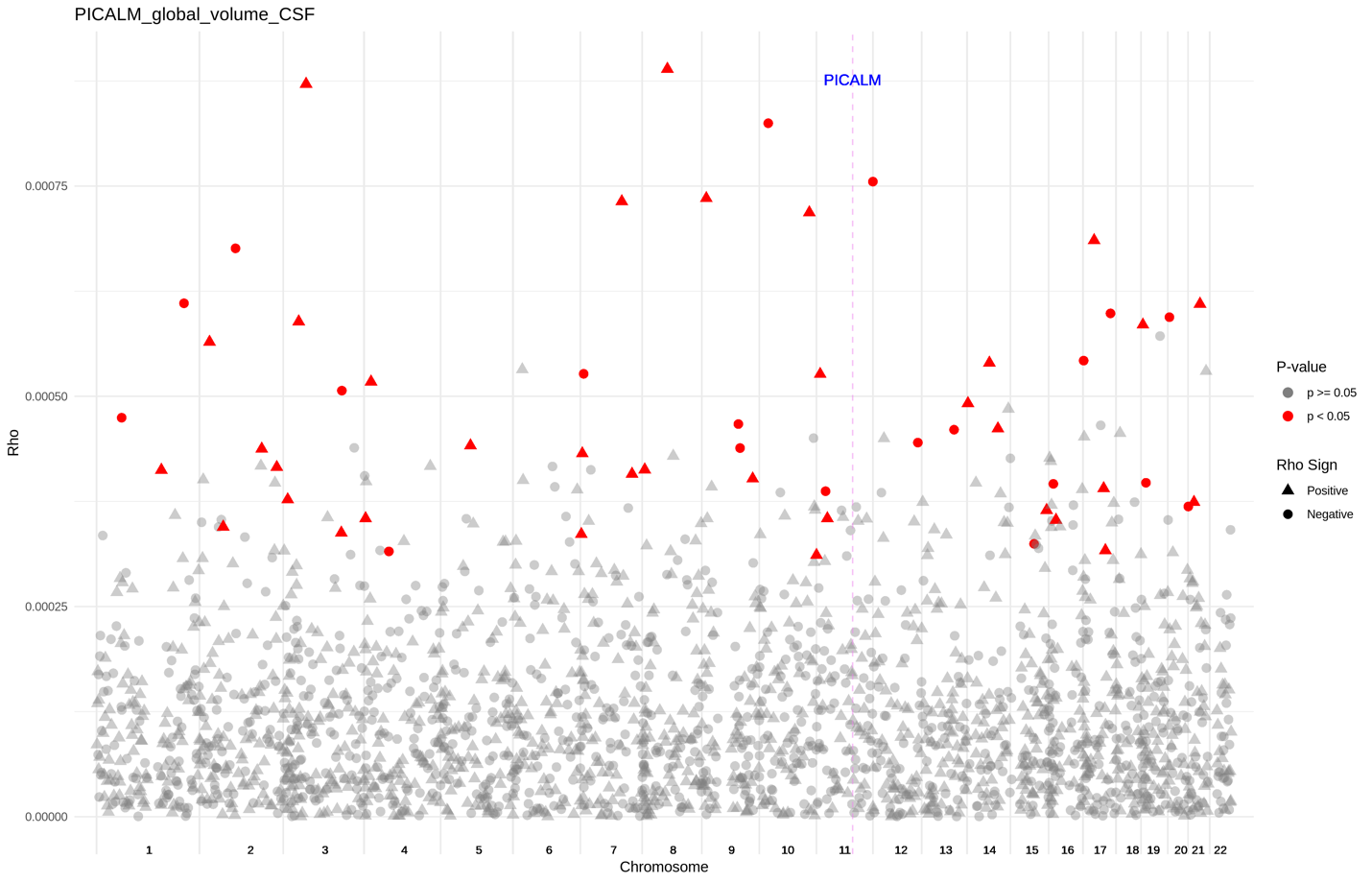


Figure S19: Genome-wide results from the local genetic covariance analysis between the family history of dementia GWAS by Marioni et al. and the listed NIDP from the image-directed analysis result prior to the MR test. Each point represents a single locus, with the y-axis location indicating the covariance between SNPs effect sizes in that region. The color of the point represents significance, with red indicating nominal significance. As each NIDP was identified according to associations with AD-genes, we overlaid the location of the associated gene onto the superGNOVA findings. Significant loci occurring within 2 megabases of the gene of interest are emphasized with a larger size.


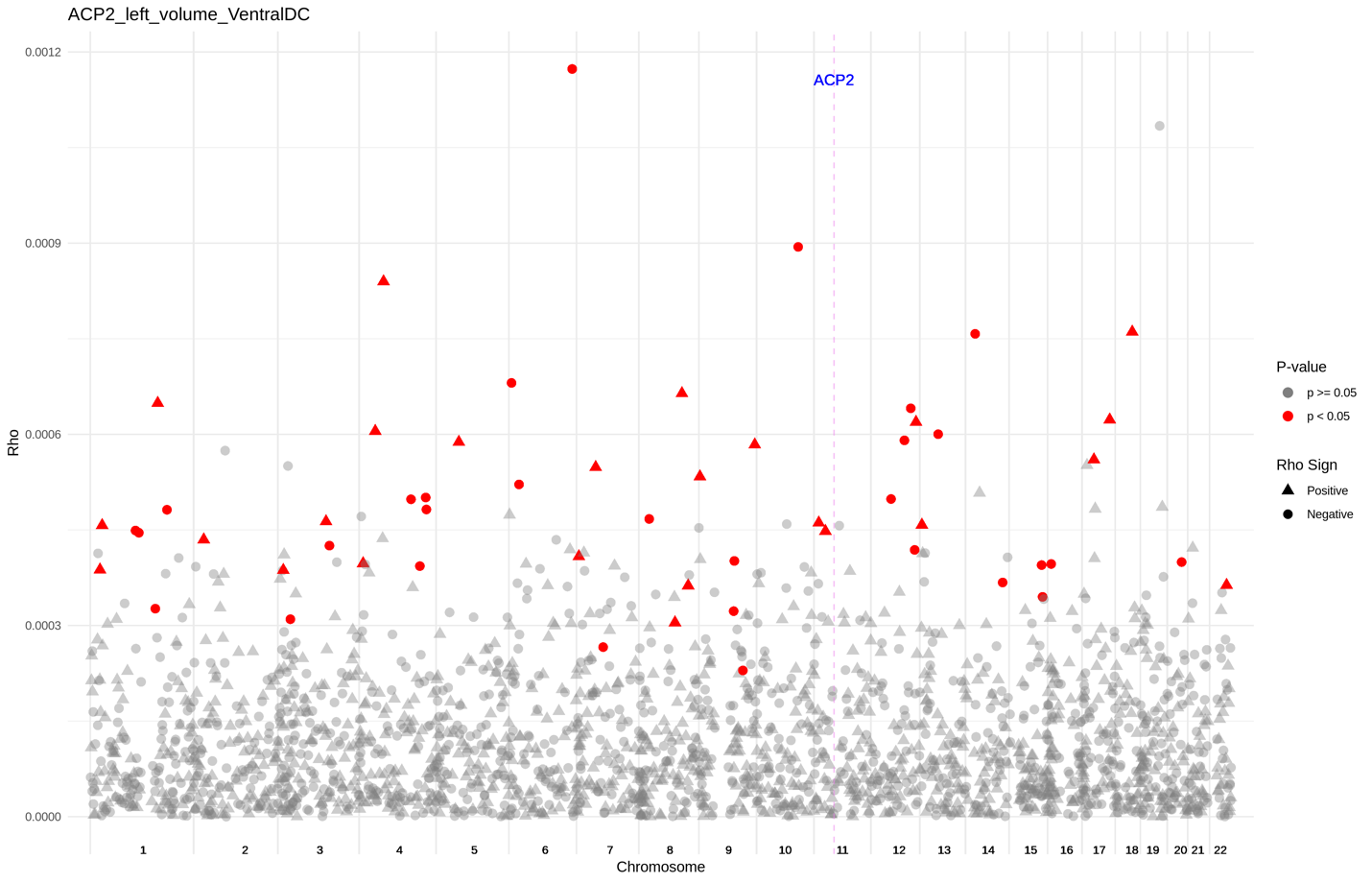


Figure S20: Genome-wide results from the local genetic covariance analysis between the family history of dementia GWAS by Marioni et al. and the listed NIDP from the image-directed analysis result prior to the MR test. Each point represents a single locus, with the y-axis location indicating the covariance between SNPs effect sizes in that region. The color of the point represents significance, with red indicating nominal significance. As each NIDP was identified according to associations with AD-genes, we overlaid the location of the associated gene onto the superGNOVA findings. Significant loci occurring within 2 megabases of the gene of interest are emphasized with a larger size.


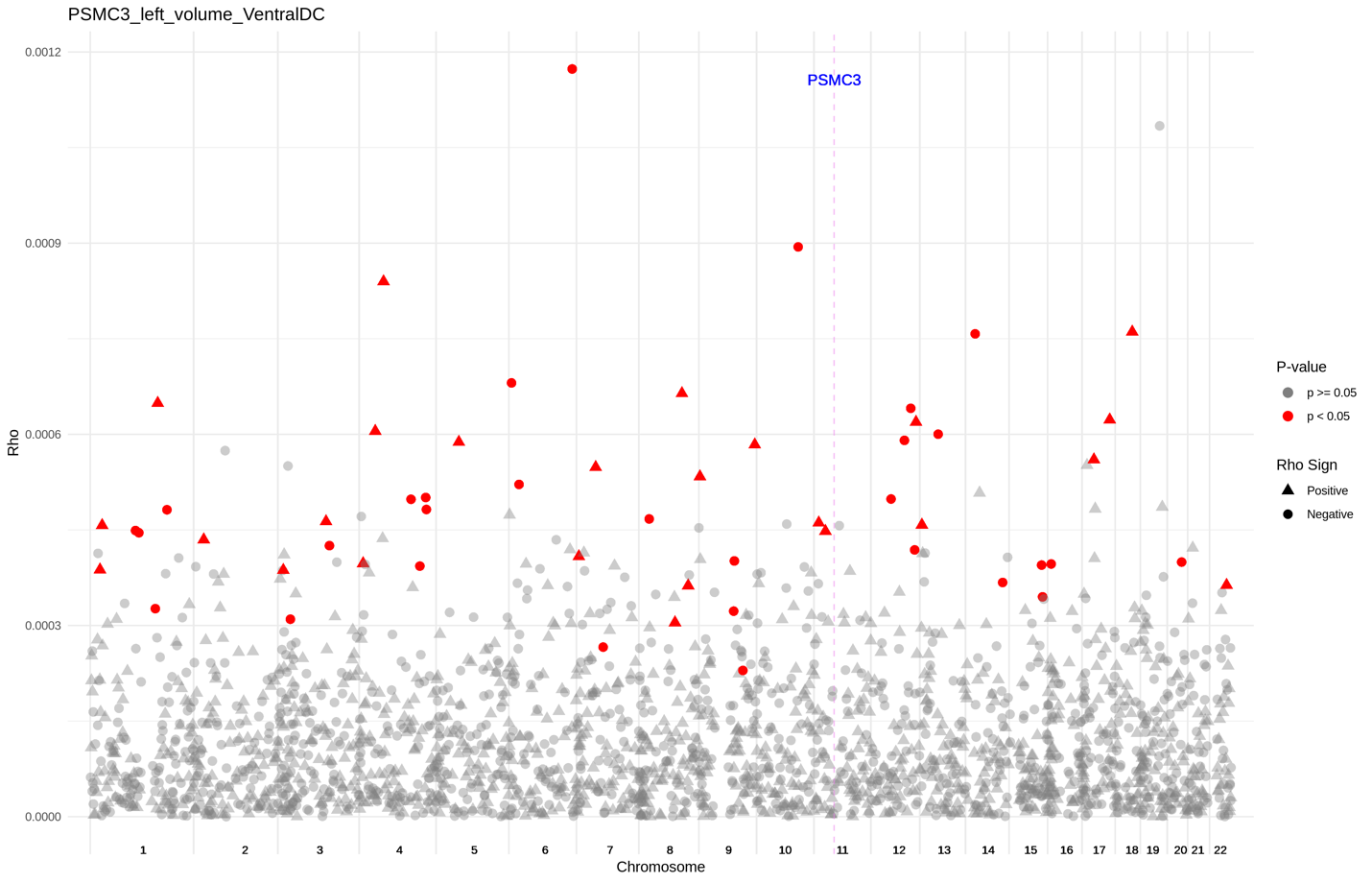


Figure S21: Genome-wide results from the local genetic covariance analysis between the family history of dementia GWAS by Marioni et al. and the listed NIDP from the image-directed analysis result prior to the MR test. Each point represents a single locus, with the y-axis location indicating the covariance between SNPs effect sizes in that region. The color of the point represents significance, with red indicating nominal significance. As each NIDP was identified according to associations with AD-genes, we overlaid the location of the associated gene onto the superGNOVA findings. Significant loci occurring within 2 megabases of the gene of interest are emphasized with a larger size.


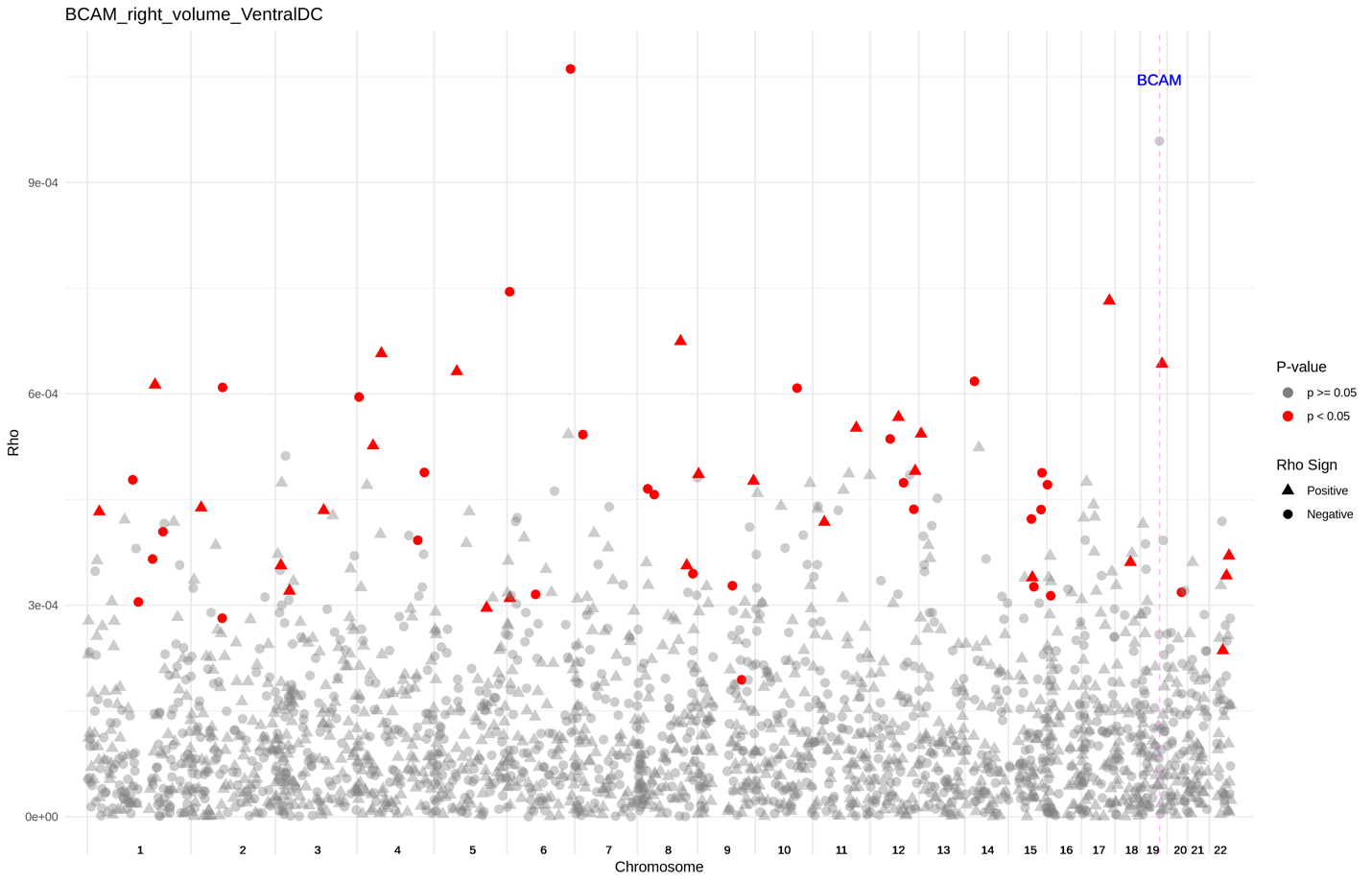


Figure S22: Genome-wide results from the local genetic covariance analysis between the family history of dementia GWAS by Marioni et al. and the listed NIDP from the image-directed analysis result prior to the MR test. Each point represents a single locus, with the y-axis location indicating the covariance between SNPs effect sizes in that region. The color of the point represents significance, with red indicating nominal significance. As each NIDP was identified according to associations with AD-genes, we overlaid the location of the associated gene onto the superGNOVA findings. Significant loci occurring within 2 megabases of the gene of interest are emphasized with a larger size.


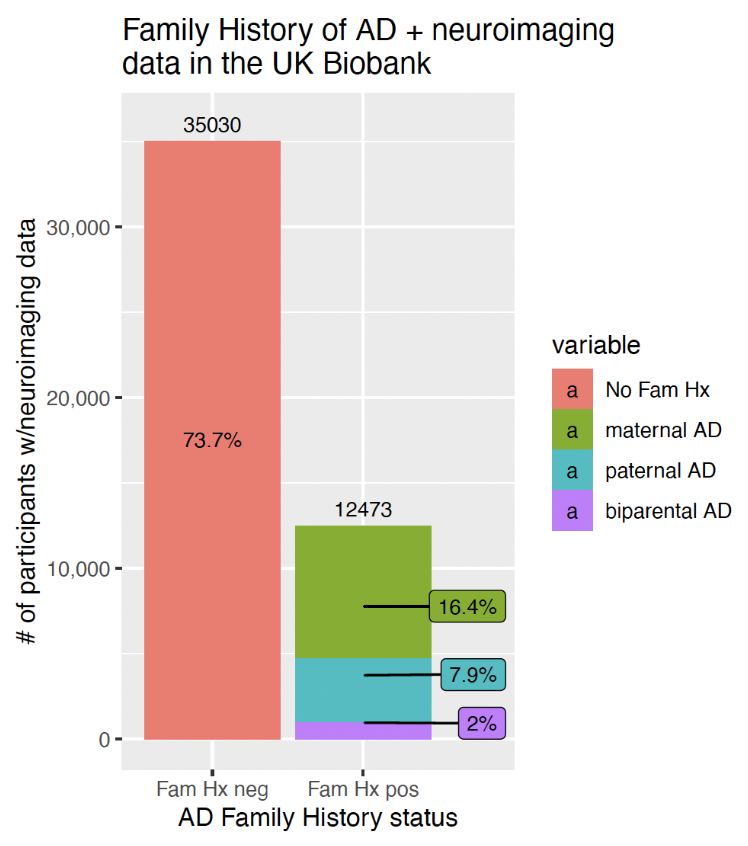


Figure S23: Distribution of individuals in the UK Biobank with and without self-reported parental history of Alzheimer’s disease and related dementias, with positive cases split according to the parent. Fam Hx – family history; AD – Alzheimer’s disease
